# Knowledge transmission patterns at the border: ethnobotany of Hutsuls living in the Carpathian Mountains of Bukovina (SW Ukraine and NE Romania)

**DOI:** 10.1186/s13002-020-00391-3

**Published:** 2020-07-10

**Authors:** Giulia Mattalia, Nataliya Stryamets, Andrea Pieroni, Renata Sõukand

**Affiliations:** 1 Department of Environmental Sciences, Informatics and Statistics, Ca’ Foscari University of Venice, Via Torino 155, 30172, Mestre, Venezia, Italy; 2 Autonomous University of Barcelona, Institute of Environmental Science and Technology, 08193, Bellaterra, Cerdanyola del Vallès, Spain; 3 University of Gastronomic Sciences, Piazza Vittorio Emanuele II 9, 12042 Pollenzo/Bra, Italy; 4 Medical Analysis Department, TISHK International University, Qazi Muhammad, Erbil, Kurdistan 44001 Iraq

**Keywords:** Biocultural diversity, Ecological Knowledge, LEK, Minority groups, TEK, Wild food plants, Wild medicinal plants

## Abstract

**Background:**

Cross-border research is a novel and important tool for detecting variability of ecological knowledge. This is especially evident in regions recently divided and annexed to different political regimes. Therefore, we conducted a study among Hutsuls, a cultural and linguistic minority group living in Northern and Southern Bukovina (Ukraine and Romania, respectively). Indeed, in the 1940s, a border was created: Northern Bukovina was annexed by the USSR while Southern Bukovina remained part of the Kingdom of Romania. In this research, we aim to document uses of plants for food and medicinal preparations, discussing the different dynamics of Local Ecological Knowledge (LEK) transmission among Hutsuls living in Ukraine and Romania.

**Methods:**

Field research was conducted using convenience and snowball sampling techniques to recruit 31 Hutsuls in Ukraine and 30 in Romania for participation in semi-structured interviews regarding the use of plants for medicinal and food preparation purposes and the sources of such knowledge.

**Results:**

The interviews revealed that, despite a common cultural and linguistic background, ethnobotanical knowledge transmission occurs in different ways on each side of the border. Family is a primary source of ethnobotanical knowledge transmission on both sides of the border; however, in Romania, knowledge from other sources is very limited, whereas in Ukraine interviewees reported several other sources including books, magazines, newspapers, the Internet and television. This is especially evident when analysing the wild plants used for medicinal purposes as we found 53 taxa that were common to both, 47 used only in Ukraine and 11 used only in Romania. While Romanian Hutsuls used almost exclusively locally available plants, Ukrainian Hutsuls often reported novel plants such as *Aloe vera*, *Aronia melanocarpa* and *Elaeagnus rhamnoides*. Knowledge related to these plants was transferred by sources of knowledge other than oral transmission among members of the same family. Therefore, this may imply hybridization of the local body of knowledge with foreign elements originating in the Soviet context which has enriched the corpus of ethnobotanical knowledge held by Ukrainian Hutsuls.

**Conclusions:**

While ethnobotanical knowledge among Romanian Hutsuls is mainly traditional and vertically transmitted, among Ukrainian Hutsuls there is a considerable proportion of LEK that is transmitted from other (written and visual) sources of knowledge. This cross-border research reveals that despite a common cultural background, socio-political scenarios have impacted Hutsul ethnobotanical knowledge and its transmission patterns.

## Background

The current global changes demand thorough analysis of not only ecological knowledge per se but also how such knowledge is produced, shared and used [1]. Indeed, ecological knowledge is a valuable system, which can significantly contribute to a better understanding of the current socio-economic and environmental changes occurring all over the word [2, 3]. These bodies of knowledge are seriously endangered by urbanization and the increasing adoption of new modes of life disconnected from local ecosystem dynamics and resources [4]. In addition, a widespread tendency of formal education (e.g. literature) to downplay local resources and knowledge has been observed [5], thus leading to knowledge homogenization and standardization [6, 7].

Ethnobotanical knowledge can be considered as part of local ecological knowledge (LEK) and it can be, but not necessarily is, regarded as traditional. Indeed, LEK ‘concerns the interplay among organisms and between organisms and their environment. LEK may be a mix of scientific and practical knowledge; it is site-specific and often involves a belief component’ [8]. Berkes [9] defined traditional ecological knowledge (TEK) as ‘a cumulative body of knowledge and beliefs, handed down through generations by cultural transmission, about the relationship of living beings (including humans) with one another and with their environment.’ As observed by Olsson and Folke [8], the specific characteristics of TEK lie in its ‘historical and cultural continuity of resource use’.

These two definitions of ecological knowledge are not in opposition, but rather, as largely accepted by the majority of ethnobiology scholars, both TEK and LEK define a complex and heterogeneous body of folk knowledge, practices and beliefs related to the natural environment. However, solely for the instructive aim of a better understanding of the different ‘nature’ of these bodies of knowledge in the current context, in this article we adopt the term TEK when referring to a system in which knowledge and practices are mainly orally transmitted (e.g. pre-industrial contexts), while we use the term LEK to refer to a system in which the borders between written (or in other words ‘standardized’) and oral forms of knowing nature and practicing this knowledge are more blurred.

Van den Boog et al. ([10] and references within) discussed and categorized the dynamics of LEK transmission into vertical (between generations within the family), horizontal (between people of the same generation) and oblique (between generations not belonging to the same family). The evolving dynamics of ethnobotanical knowledge transmission have been found to be affected by not only exposure to a market economy [11, 12] but also socio-economic changes [13] and political circumstances [7].

Hutsuls are an ethnic group living in the Carpathian Mountains of Ukraine and to a lesser extent Romania. These communities have been recently studied from an ethnographic perspective [14, 15] as well as an ethnobotanical one [6, 16].

**Fig. 1:**
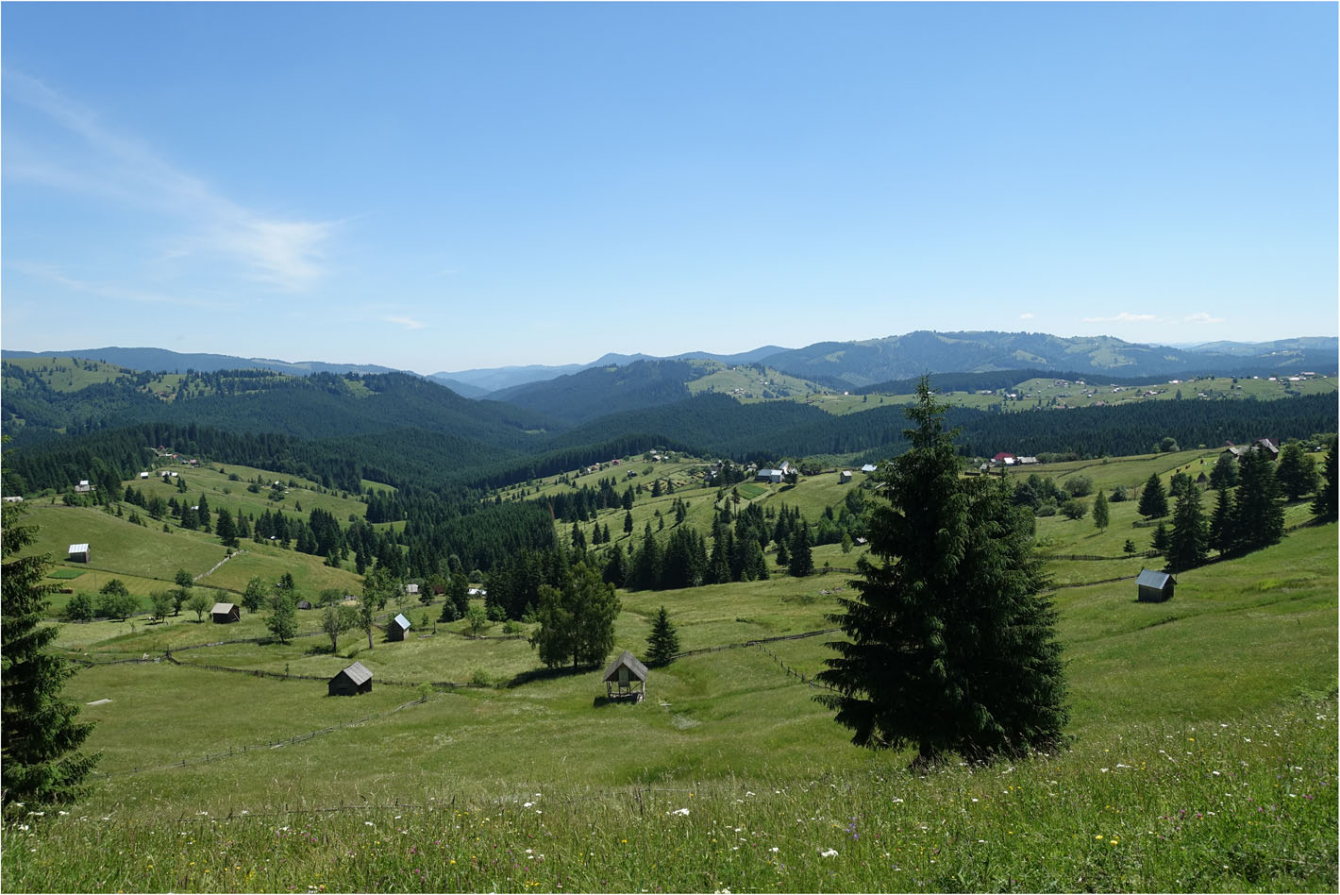
Bukovinian Carpathian landscape, Lupcina, Romania; July 2019; Photo by N. Stryamets

Over the last few years, cross-border ethnobotany has received increasing attention from scholars [16–18] as it is an excellent tool for exploring the effects of different social and political contexts on LEK. In this study, we examine culturally homogenous Hutsul communities living in similar mountain landscapes (Fig. 1), yet separated by a border created in the 1940s when Northern Bukovina was annexed by the Soviet Union and Southern Bukovina remained part of the Kingdom of Romania. The aim of this study, therefore, is to document and discuss the different dynamics of LEK transmission among Hutsuls living in Ukraine and Romania and to explore whether the different social, political and economic conditions that developed after border creation have affected these dynamics.

## Methods

### Study area and historical background

**Fig. 2:**
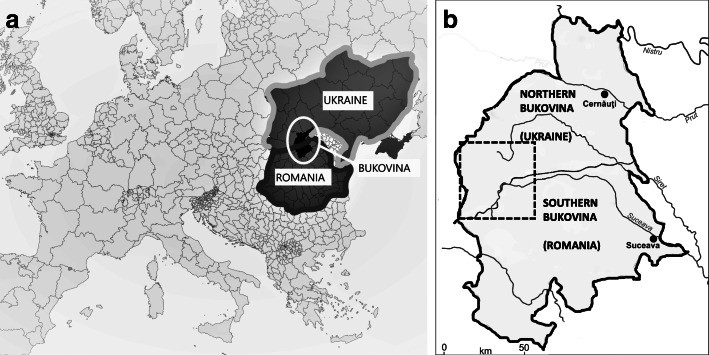
**a***,***b** Maps of the study area

The interviews were conducted in Bukovina (Fig. 2), a region of Eastern Europe characterized by an extensive forested area especially in proximity to the Carpathian Mountains. This region belonged to the Hapsburg Empire for over 140 years until 1918, when it became part of the new Kingdom of Romania. In 1940, the Ribbentrop Molotov Pact split this region into two parts: Northern Bukovina was annexed by the USSR and thus a new border was created. After a few years of uncertain borders, in 1944 Southern Bukovina was assigned to Romania, and since 2007 it has been a member of the European Union, whereas Northern Bukovina, after the collapse of the Soviet Union in 1991, became part of independent Ukraine. While Northern Bukovina underwent a process of homogenization and centralization promoted by the USSR, Southern Bukovina was not heavily affected by Romanian collectivization policies due to its limited economic interest.

Over one century ago, Bukovina was a multicultural and multi-religious mosaic composed of Romanians, Ukrainians, Jews, Armenians, Roma people, Hungarian Székelys, Russian Old Believers (Lipovans), Germans (mainly clerks), Slovaks, Poles and Tatars [19, 20].

Currently, only a small portion of such cultural diversity is maintained, as linguistic and ethnic minorities have undergone a process of homogenization [21]. Among these minorities are Hutsuls who live in the Carpathian Mountains of the Suceava district of Romania and the Cernivtci, Ivano-Frankivs’k and Zakarpatska provinces of Ukraine. Hutsuls speak a local language which they themselves consider to include elements of Ukrainian, Polish, German and Hungarian [14]. In Romania, children are taught both in Romanian and Ukrainian in school, while at home they mainly speak the Hutsul language. In Ukraine, they attend school in Ukrainian and use Hutsul for informal conversations. The main economic activities of both Romanian and Ukrainian Hutsuls are small-scale mixed farming and non-wood forest product exploitation. All interviewed Hutsuls belonged to the Orthodox Church.

The climate of the area is classified as Dfb, a humid continental climate, without a dry season and with warm summers. Annual precipitation is around 775 mm, which is mainly concentrated in June and July. The coldest month is January when average temperature is − 5.5 °C and the warmest is July at 16 °C.

### Sampling and interviews

**Fig. 3:**
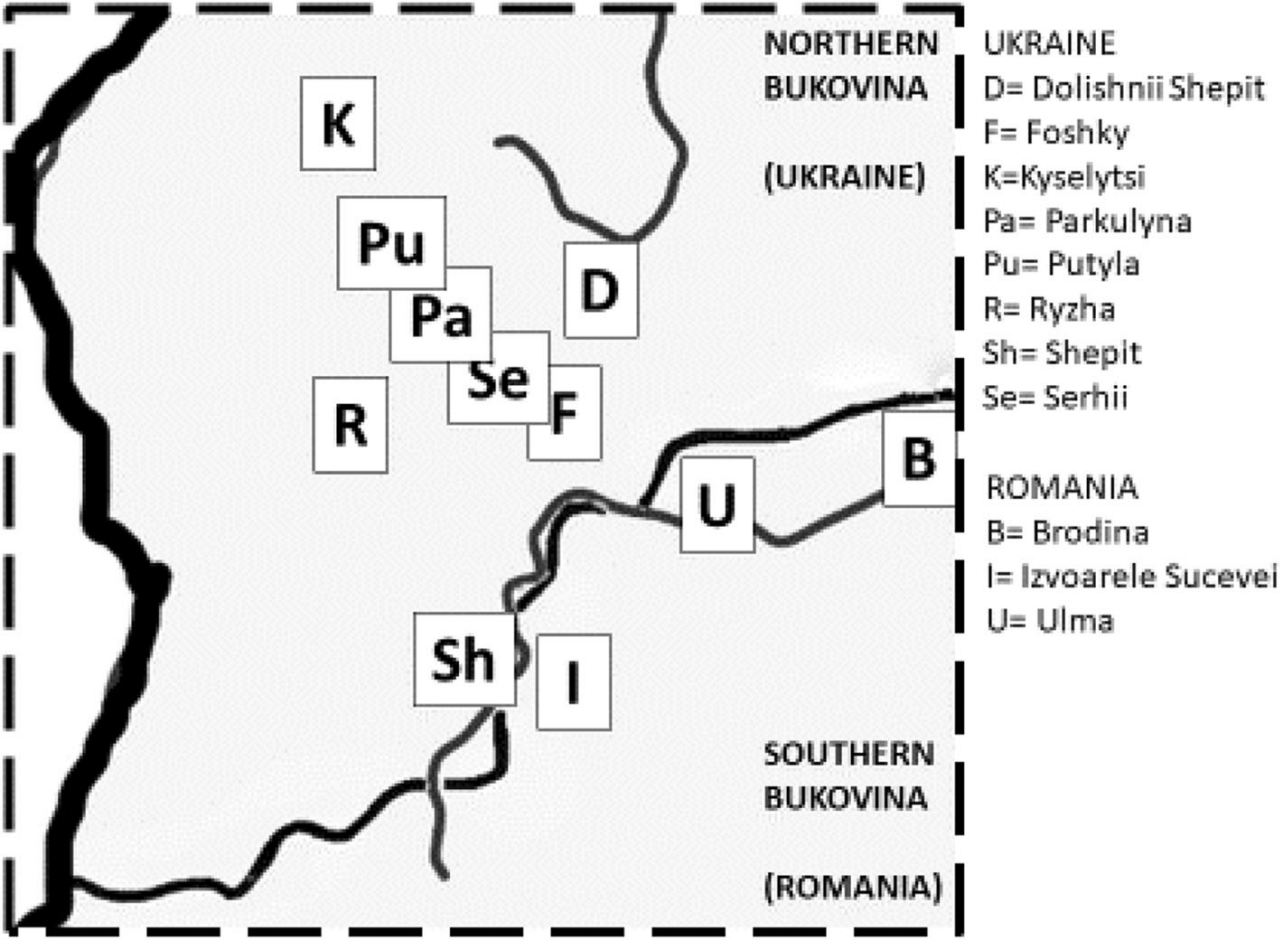
Map of the specific study area

Sixty-one Hutsul informants were interviewed in Romanian and Ukrainian Bukovina between June 2018 and July 2019. Thirty in-depth interviews where gathered in the municipalities of Brodina, Ulma and Izvoarele Sucevei, in the district of Suceava, Northern Romania, while 31 interviews were conducted in the districts of Putyla (main villages in which interviews were conducted include Kyselytsi, Shepit, Serhii, Foshky, Parkulyna, Ryzha) and Vyzhnytsia (Dolishnii Shepit) in the province of Cernivtci, Southern Ukraine (Fig. 3). Altitude of the villages ranges from 600 to 1000 m a.s.l.

Informants were conveniently selected (we interviewed people walking on the street, working in their gardens or talking in cafes) and when possible we used the snowball method. We strictly followed the ethical guidelines prescribed by the International Society of Ethnobiology (ISE 2006) and the study protocol was approved by the Ethical Committee of Ca’ Foscari University of Venice.

We used semi-structured interviews to obtain qualitative and quantitative data regarding the use of plants for culinary and medicinal purposes (starting with culinary use). Specifically, we asked interviewees what plants they harvest, for what purpose and how they prepare them. In addition, we asked informants the source of such knowledge, i.e. from whom or where did they learn it. In some cases, we deemed it useful to draw a timeline indicating when each informant started using each plant. When possible, we harvested the mentioned plants together with the interviewees in order to collect and identify voucher specimens. Voucher specimens collected in Ukraine are stored in the ‘Roztochya’ Nature Reserve (Ukraine) bearing codes NB001–NB259, while those collected in Romania are stored in the Herbarium of Ca’ Foscari University of Venice (Italy) bearing codes SB001–SB094. Voucher specimens were identified using the ‘The Plant List’ [22] and ‘Flora Europaea’ [23]. Plant families were classified according to Stevens [24]. Interviews were held in Romanian or Ukrainian according to the preference of the interviewees. In Romania, many interviewees answered using a mixture of Romanian, Ukrainian or the Hutsul language, while in Ukraine they tended to stick to Ukrainian.

### Data analysis

Gathered data on the use of plants for various purposes were entered into an Excel spreadsheet. We structured emic categories into detailed use-reports (DUR), where each interviewee mentioned a specific use (e.g. abdominal pain) of a plant part (e.g. aerial parts or roots) for a specific preparation (e.g. tea or infused in alcohol). The spreadsheet included informant code, language of the interview, plant parts used, scientific name, family assignment, local name (Ukrainian and Hutsul names were transliterated using the system adopted by the Cabinet of Ministers of Ukraine [25]), mode of preparation, time of use (always, in the past, recently abandoned, recently adopted), medicinal use, food use, source of knowledge and comments. In addition, for medicinal uses, we related ICD-11 medicinal categories [26] to reported emic categories (e.g. good for the stomach) for better comparison. In addition to the ICD-11 (International Classification of Diseases) categories, we included a general health category including mainly ‘general symptoms’ and some unspecified emic categories.

We considered only wild plants for food purposes, while we also included cultivated plants for medicinal purposes. We considered as ‘wild’ plants that grow without intended cultivation. This category mainly consists of native and naturalized species, but also plants not cultivated for food or medicinal purposes (e.g. *Tilia cordata*), as well as species that are generally gathered from the wild but can also be cultivated (e.g. *Rubus idaeus*).

To compare Romanian and Ukrainian Hutsuls, we calculated the Jaccard Similarity Index (JI) following the methodology of González-Tejero et al. [27]:

$$ \mathrm{JI}=\left(C/\left(A+B-C\right)\right)\times 100 $$

where *A* is the number of species in sample A, *B* is the number of species in sample B and *C* is the number of species common to both A and B. An index value close to 100 indicates that the two groups are very similar, while a value close to 0 indicates that are very different.

In order to calculate the proportion of each knowledge transmission strategy, we assigned a total of 1 point to each source of knowledge indicated by the interviewee. Therefore, if the interviewee reported one source (e.g. parents), we assigned a value of 1; two sources (e.g. books and grandparents), we assigned 0.5 to each; three sources, 0.33 to each; and four sources, 0.25 to each. Later, we summed these values in the emic categories of knowledge source mentioned by the interviewees on both sides of the border.

## Results and discussion

We recorded a total of 118 food and medicinal plants from 107 genera and 53 families. The most well represented families were Asteraceae, Rosaceae and Lamiaceae. Among Hutsuls of Northern Bukovina, we recorded 107 taxa, while there were 72 taxa among Hutsuls of Southern Bukovina, and 60 taxa common to both. The most used plants were *Vaccinium myrtillus*, *Rubus idaeus* and *Urtica dioica.* These were the most used in both Northern and Southern Bukovina and thus we can confirm their importance as Hutsul cultural markers as previously suggested by Sõukand and Pieroni [16].

### Food taxa

**Fig. 4:**
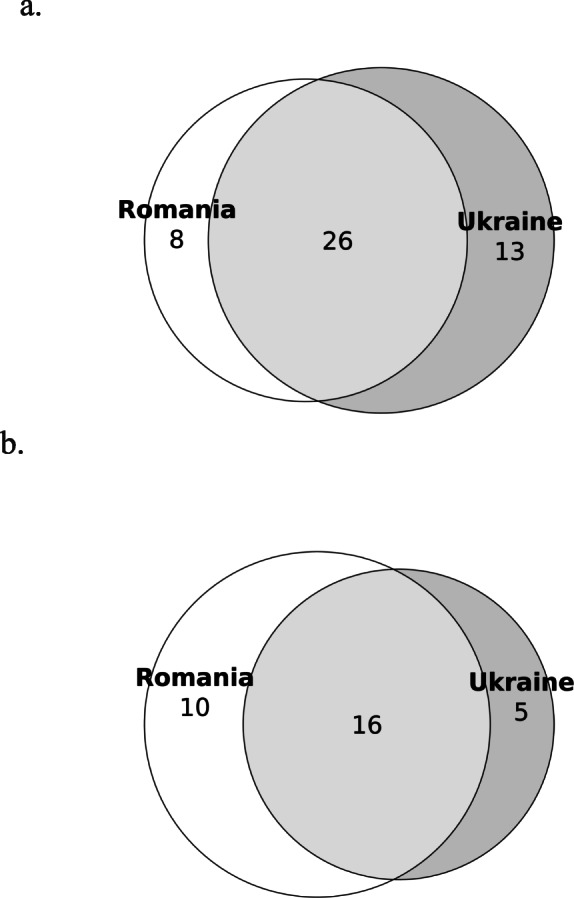
**a** The proportional Venn diagram shows that most of the food taxa mentioned are common to Hutsul communities of Northern and Southern Bukovina; JI = 55. **b** The proportional Venn diagram of food taxa mentioned by at least three interviewees shows that Romanian Hutsuls reported more consistent uses than Ukrainian Hutsuls. Indeed, several food taxa were mentioned by only one or two Ukrainian Hutsuls; JI = 51

The interviewed Hutsuls reported a total of 47 taxa used for food preparations (Table 1). Twenty-six taxa were found on both sides of the border, eight taxa were reported only in Romania and 13 only in Ukraine (Fig. 4). When considering only the plants mentioned by at least 10% of the interviewees (three), Romanian Hutsuls mentioned ten taxa, Ukrainians five taxa and 16 were common to both. The Jaccard Similarity Index (JI) for the two groups was 55 when based on all the taxa, while an index value of 51 was observed when considering only the taxa mentioned by 10% of interviewees. The most common taxa correspond to those most used overall (*Vaccinium myrtillus*, *Rubus idaeus* and *Urtica dioica*), although among Romanian Hutsuls, *Fagus sylvatica* was also very common as its wood was used for smoking pork meat, which is one of the most traditional Hutsul preparations, as well as to flavour soups. *Rumex acetosa* was very often reported by Ukrainian Hutsuls (but never by Romanian Hutsuls) as an ingredient for soups. The most common food purpose was recreational tea, a preparation used for 30 taxa. Tea was widely reported in Northern Bukovina where 23 taxa were mentioned, of which 13 were shared with Southern Bukovina, for a total of 81 DUR. In addition, six taxa were reported only among Hutsuls in Southern Bukovina for a total of 19 taxa and 65 DUR. In line with Sõukand et al. [28], the main represented families for recreational teas were Rosaceae followed by Asteraceae and Lamiaceae. Another common preparation was jam which predominated in Romania (82 DUR) and included eight taxa, five of which were common to both communities (*Fragaria vesca*; *Rubus caesius*; *Rubus idaeus*; *Vaccinium myrtillus*; *Vaccinium vitis-idaea*). Among the taxa used for jams exclusively prepared by Romanian Hutsuls, we found the young sprouts of *Picea abies*, which are harvested in June and can also be used for making medicinal syrup, and the petals of *Rosa rugosa* and *Rosa centifolia*, which are used for jams and teas almost exclusively by Romanian Hutsuls. In addition, the flowers of *Taraxacum officinale* were also used for the preparation of jam in Southern Bukovina. Another common use of wild food plants was seasoning, and in particular *Thymus* spp. and *Armoracia rusticana* which were used in both communities. Actually, *Armoracia rusticana* was reported by Ukrainian Hutsuls for ‘квашення’ (kvashennya), which is a lacto-fermented preparation of cucumbers, tomatoes, cabbage or other vegetables, a typical recipe common in Romania under the name of ‘muraturi’. For this preparation, Hutsuls from both communities reported the use of *Armoracia rusticana* roots (in Ukraine leaves were also reported) and *Quercus* spp. (young branches in Romania and leaves in Ukraine), and *Carum carvi* only in Ukraine. Many other cultivated plants (cucumbers, carrots, garlic, cabbage, cauliflower, as well as mushrooms in some cases) were added to this preparation, which is later fermented. Another peculiar mode of preparation reported in Ukraine is ‘*Квас* (*kvas*)’, a drink made from fermented grain and low in alcohol content. Birch sap was also reported as an ingredient for kvas. Such a drink is often flavoured with berries and fruits, including *Aronia melanocarpa*, *Sorbus* spp. and *Vaccinium vitis*-*idaea* which were mentioned by interviewees.

**Table 1:** Recorded food taxa in Northern and Southern Bukovina. *DUR* Detailed Use Reports; *RO* Romanian Hutsuls; *UA* Ukrainian Hutsuls

Latin name, Family and voucher specimens	Local names	Used part(s)	Preparation	DUR	
				RO	UA
*Acer* spp. including *Acer pseudoplatanus* L. (Sapindaceae) NB225; NB226	Paltin; явір; клен (Yavir; klen)	Sap	Drink		4
		Fruits (dried)	Tea	4	
*Achillea millefolium* L. (Asteraceae) SB011; SB050; SB074 NB007; NB017; NB039	Coada șoaricelului; деревій (Derevii)	Aerial parts (dried)	Tea	3	2
*Armoracia rusticana* P.Gaertn., B.Mey. & Scherb. (Brassicaceae) SB031 NB028	Hrean; хрень; хрін; хреню (Khren; khrin; khreniu)	Roots	Salad (with beetroots)	13	3
			Seasoning	7	
			Raw		5
			Pickles (cucumbers, tomatoes)		7
			Fermenting		1
		Leaves	Pickles (cucumbers)	1	
		Whole plant	Seasoning	1	
*Arnica montana* L. (Asteraceae)	Гарник; арник (Harnyk; arnyk)	Aerial parts (dried)	Tea	2	3
*Aronia melanocarpa* (Michx.) Elliott (Rosaceae)	чорна рябіна (Chorna riabina^)	Fruits	*Kvas*		1
*Atriplex hortensis* L. (Amaranthaceae) SB004; SB018	Lobodă; натина§; лобода (natyna§; loboda)	Aerial parts	Soup	8	
		Leaves	*Sarmale*	2	
*Betula pendula* Roth (Betulaceae) NB041; NB049; NB115	Береза (bereza)	Sap	Drink		11
			Strong alcohol		5
		Leaves	Mixed tea		1
*Carum carvi* L. (Apiaceae) SB007 NB037	Săcărică; Cmin; Hmel; хміль§; тмин; хміль польовий§; кмин (Khmil§; tmyn; Khmil polovyi§; kmyn)	Aerial parts	Tea	3	
			Seasoning	2	
		Seeds	Tea	1	5
			Seasoning	2	
			Fermenting	3	
			Pickles		2
			Bread additive		3
*Chenopodium album* L. (Amaranthaceae) SB022 NB139	Lobodă; натина§; лебеда (Natyna§; lebeda)	Aerial parts	Soup	3	2
			Stewed (with cream)	1	1
			Seasoning (dried)	1	
*Cichorium intybus* L. (Asteraceae)	петрів батіг (Petriv batih)	Aerial parts	Tea		1
*Coriandrum sativum* L. (Apiaceae)	колєндра (Koliendra)	Seeds	Smoking (meat seasoning)		1
*Corylus avellana* L. (Betulaceae) SB089	Alune	Fruits	Raw	5	
*Crataegus* spp. including *Crataegus monogyna* Jacq. (Rosaceae) NB006; NB066	Глід (Hlid)	Fruits	Tea		2
*Epilobium angustifolium* L. (Onagraceae) NB057	іван чай, демник§; ;имник§ (ivan chai, demnyk§; dymnyk§)	Aerial parts	Tea		3
*Equisetum* spp. including *Equisetum arvense* L.; *Equisetum sylvaticum* L (Equisetaceae) SB020 NB068, NB093, NB113, NB114	Barba ursului; Coada calului; Padivolos; хвощ польловий, падиволос§; (Khvoshch pollovyi, padyvolos)	Aerial parts	Tea	2	1
*Fagus sylvatica* L. (Fagaceae) SB060, NB168	Fag; бук (Buk)	Wood	Smoking (meat)	19	1
*Fragaria vesca* L. (Rosaceae) SB094 NB004, NB015, NB056	Fragi; Frăguța; ягода§; ягоди; ягода черлена§; (yahoda§; yahody§; yahoda cherlena§)	Fruits	Raw	4	2
			Jam	14	9
			Compote	3	1
			Dessert	2	
			Tea	2	
			Juice	1	1
			Syrup	2	
			Frozen		1
*Gentiana* spp. possibly including *Gentiana lutea* L. and *Gentiana asclepiadea* L. (Gentianaceae)	Gingiura	Aerial parts	Infused in strong alcohol	4	
*Humulus lupulus* L. (Cannabaceae) SB081 NB182	Hamei; хміль (Khmil)	Flowers	Beer	6	
			Bread starter		2
*Hypericum* spp. including *Hypericum perforatum* L. (Hypericaceae) SB068; SB092 NB005, NB034, NB046, NB085	Pojarniță; Sunătoare; звіробій; звіробой (Zvirobii; zviroboi)	Aerial parts	Tea	3	8
*Levisticum officinale* W.D.J.Koch (Apiaceae)	Любисток (Liubystok)	Aerial parts	Tea		2
*Matricaria chamomilla* L. (Asteraceae) SB002; SB019 NB127	Mușețel; Romaniță; ромашка; романіца; романець; румєниць (Romashka; romanitsa; romanets; rumienyts)	Aerial parts	Tea	4	9
*Mentha* spp. (Lamiaceae) SB014; SB016; SB034; SB096 NB079, NB080, NB097	Mentă de doi culoari; менти; Mentă tare; Minta; мята (Miata)	Leaves	Tea	5	1
*Origanum vulgare* L. (Lamiaceae) NB033; NB055; NB021	Материнка (Materynka)	Aerial parts	Tea		4
			Seasoning		1
*Oxalis acetosella* L. (Oxalidaceae) NB058	Квасениця звичайна (Kvasenytsia zvychaina)	Leaves	Salad		1
			Snack		1
*Papaver rhoeas* L. SB044a; SB044b; SB044c	Mac	Seeds	Food additive	1	
*Picea abies* (L.) H. Karst. (Pinaceae) SB008; SB021 NB043	Brad; смерека (Smereka)	Sprouts	Jam	2	
		Wood	Smoking (meat)	1	2
*Plantago major* L. (Plantaginaceae) NB022; NB047; NB132	Подорожник (Podorozhnyk)	Aerial parts	Tea		2
*Populus tremula* L. (Salicaceae)	Осика (Osyka)	Wood	Smoking (meat)		1
*Primula veris* L. (Primulaceae)	Cioboțica cucului	Aerial parts	Tea	6	
*Quercus* spp. including *Quercus robur* L*.* and *Quercus rubra* L. (Fagaceae) SB056 NB160	Stejar; Duba; дуб (Dub)	Leaves	Pickles (cucumbers)		3
		Young branches	Pickles	6	
*Rosa canina* L. (Rosaceae) SB062 NB016; NB083	Măceș	Fruits	Tea	1	
*Rosa rugosa* L*.; Rosa centifolia* L. (Rosaceae) SB023	Trandafir; роза (Roza)	Petals	Jam	14	
			Jelly	2	
			Syrup	4	
			Tea	4	
*Rubus* spp. including *Rubus caesius* L. and *Rubus fructicosus* L. (Rosaceae) SB083 NB010; NB062;NB063	Чорниця; ожина; єжевіка (Chornytsia; ozhyna; yezhevika) Mure; чорниці (Chornytsi)	Fruits	Jam	9	3
			Raw	2	
			Compote	1	1
			Infused in alcohol	2	
			Juice	1	1
			Syrup	2	
		Aerial parts	Tea	2	
		Flowers	Tea		1
*Rubus idaeus* L. (Rosaceae) SB009; SB071 NB081	Zmeură; малина (Malyna)	Aerial parts	Tea	3	4
		Fruits	Juice	5	6
			Raw	7	2
			Compote	6	7
			Jam	18	13
			Dessert	1	
			Syrup	4	
			Frozen		2
			Tincture		1
*Rumex acetosa* L. (Polygonaceae) NB081	Квас§; щавель; квасок§ (kvas§; shchavel; kvasok§)	Leaves	Soup		21
			Salad		2
			Snack		2
*Rumex alpinus* L. (Polygonaceae) SB067 NB003	Ștevie	Leaves	Stewed (with cream)	1	
*Sambucus nigra* L. (Adoxaceae) SB084 NB054	Soc; бузина ( Buzyna)	Flowers	Juice	2	
			Tea	1	
		Fruits	Jam		2
*Sorbus* spp. including *Sorbus aucuparia* (Rosaceae) SB055 NB232	Scoruș; щкорох§ (shchkorokh§)	Flowers	Tea		1
		Fruits	*Kvas*		1
			Various	4	
*Taraxacum officinale* Webb (Asteraceae) SB063 NB016; NB048	Papădie; кульбаба (kulbaba)	Flowers	Jam	1	
		Aerial parts	Salad		3
		Roots	Salad		2
			Coffee		2^a^
			Tea		1
*Thymus* spp. including *Thymus vulgaris* L. and *Thymus serpyllum* L. (Lamiaceae) SB001; SB090 NB027; NB125; NB030	чабер; чебрець;чебрик; городній чебрець (Chaber; chebrets; chebryk; horodnii chebrets ) *Thymus vulgaris*: Cimbru; Cimbru sălbatic; чеберецьсадовий (cheberets sadovyi) *Thymus serpyllum*: Cimbrișor; чебрек польовий; чебрець звичайний, чебрик польовий; польовий чебрець (chebrek polovyi; chebrets zvychainyi, chebryk polovyi; polovyi chebrets)	Aerial parts	Tea	4	8
			Seasoning	16	8
*Tilia cordata* Mill. (Malvaceae) SB017 NB253	Tei; липа (Lypa)	Flowers	Tea	2	12
*Tussilago farfara* L. (Asteraceae) SB065; SB085 NB072; NB133	Podbal; мати й мачуха (Maty y machukha)	Leaves	*Sarmale*	5	
		Aerial parts	Tea		2
*Urtica dioica* L. (Urticaceae) SB088, NB026; NB048	Urzică; кропива (Kropyva)	Aerial parts (young)	Soup	17	
			*Borsh*	4	25
			Stewed (with cream)	2	
			Salad		1
			Seasoning		1
*Vaccinium myrtillus* L. (Ericaceae) SB006 NB060	Afina; афини; афинник (Afyny; afynnyk)	Aerial parts	Strong alcohol (*afinata*)	6	
			Tea	9	7
		Fruits	Juice	5	2
			Syrup	3	3
			Frozen	1	2
			Preserved in *rachiu*	1	
			Raw (with sugar)	2	1
			Compote	4	4
			Jam	17	18
			Cake	1	
			Preserved with sugar	1	
			‘Wine’	1	1
			Dessert		3
			Dried		1
			Snack		1
*Vaccinium vitis-idaea* L. (Ericaceae) SB010 NB061	Merișoare; ґоґодзи§; гогдзі§; брусніка (Gogodzy§; hohdzi§; brusnika)	Fruits	Raw	4	
			Jam	7	9
			Juice	8	1
			Drink	1	
			Compote	2	1
			Syrup		2
			Frozen		1
			Snack		1
			Tea		3
			Kvas		1
*Viburnum opulus* L. (Adoxaceae) NB223	Călină; калина (Kalyna)	Fruits	Strong alcohol (*Calinata*)	4	
			Preserved in jars	2	
			Syrup	2	
		Aerial parts	Tea		3

Plant names mentioned by Ukrainian Hutsuls are reported in Cyrillic (with transliteration). Plant names mentioned by Romanian Hutsuls are reported in the Latin alphabet. Plant names not reported in Romanian or Ukrainian dictionaries or in publications available for the area (e.g. Pieroni and Soukand, 2017), and are therefore probably Hutsul names, are marked with a §. Russian names are marked with a ^

^a^denotes a past use

On both sides of the border, berries were often prepared as compote, which is made by boiling fruits (in this case *Fragaria vesca*, *Rubus idaeus*, *Rubus caesius*, *Vaccinium myrtillus*, *Vaccinium vitis-idaea*) in abundant water and later removing them to drink the flavoured liquid. Berries are either eaten as a dessert or thrown away. The compote can be prepared with or without adding sugar (e.g. *Vaccinium myrtillus* compote). Compote was often reported as a preserve for winter time.

Freezing as a conservation method was mentioned only by one person in Romania (for *Vaccinium myrtillus*), while it was more often reported in Ukraine for other berries (*Rubus idaeus*, *Fragaria vesca* and *Vaccinium vitis*-*idaea*).

### Medicinal taxa

**Fig. 5:**
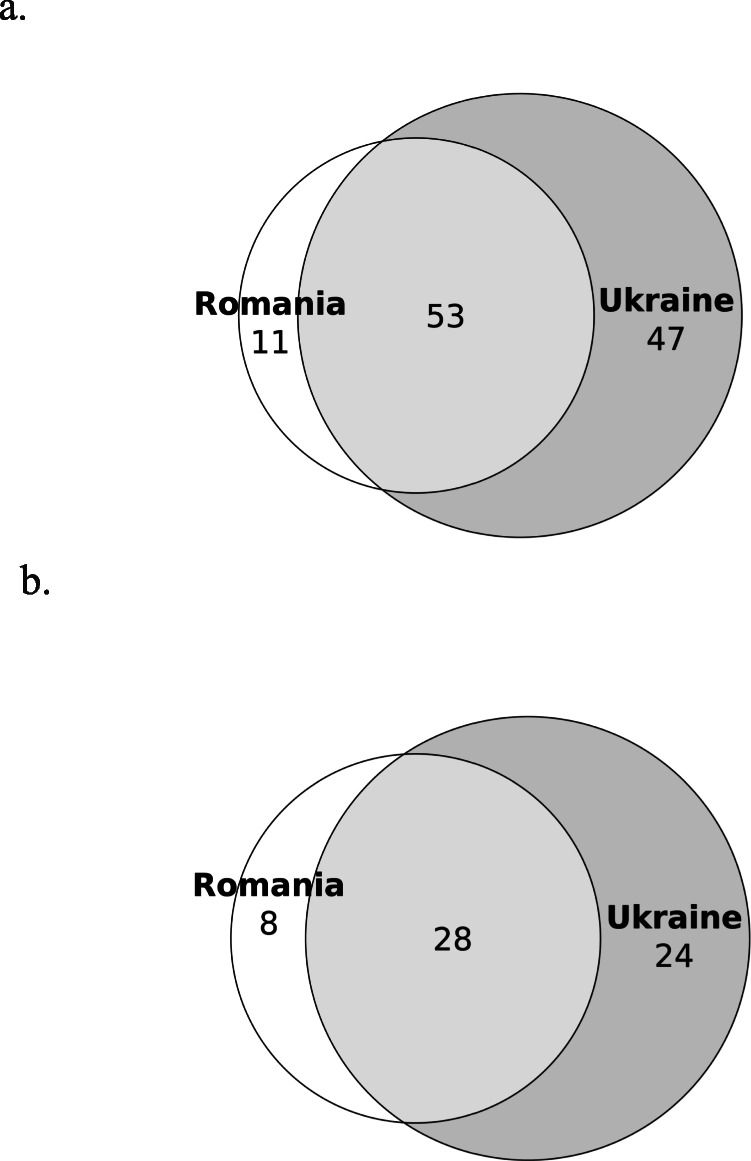
**a** The proportional Venn diagram shows that the majority of mentioned medicinal taxa were common to Hutsuls living in Northern and Southern Bukovina. However, a very large number of taxa were reported only in Ukraine; JI = 48. **b** The proportional Venn diagram of medicinal taxa mentioned by at least three interviewees shows that Romanian Hutsuls used medicinal plants more consistently than Ukrainian Hutsuls. Indeed, 23 taxa were mentioned by only one or two Ukrainian Hutsuls. This result is in line with the findings regarding the use of food taxa.; JI = 46

We recorded 111 plant taxa used for medicinal purposes (Table 2). Specifically, 64 taxa were used among Romanian Hutsuls while 100 were used among Ukrainian Hutsuls, with 53 taxa shared in common (Fig. 5). This disparity was also reflected in the number of DURs: 840 in Northern Bukovina and 585 in Southern Bukovina (− 30%). This trend was also reported by Sõukand and Pieroni [16]. The Jaccard Similarity Index did not vary much when considering all taxa (48) or only those mentioned by at least 10% of the interviewees (46).

**Table 2:** Recorded medicinal taxa in Northern and Southern Bukovina. *DUR* Detailed Use Reports; *RO* Romanian Hutsuls; *UA* Ukrainian Hutsuls

Latin name, family and voucher specimens	Local names	Used part(s)	Preparation	Medicinal Use	DUR	
					RO	UA
*Abies alba* Mill. possibly including *Picea abies* (L.) H. Karst. (Pinaceae)	Molid; ялина (Yalyna)	Resin	Locally Applied	Joint pain		2
		Young sprouts	Syrup (fresh)	Fever	1	
				Cough	2	
				Good for lungs	5	
*Achillea carpatica* Blocki ex Dubovik (Asteraceae)	Деревій карпатський (Derevii karpatskyi)	Aerial parts	Tea (dried)	Digestive system problems		1
				Stomach diseases		1
*Achillea millefolium* L. (Asteraceae) SB011; SB074; SB050 NB007; NB017; NB039	Coada șoaricelului; деревій; деревій, тисячолітник; деревій звичайний; деревій буковинський (Derevii; derevii, tysiacholitnyk; derevii zvychainyi; derevii bukovynskyi)	Aerial parts	Tea	Vessel cleansing		1
			Locally applied (juice of pressed leaves)	Wounds		3
			Tea	Diarrhoea		3
				Digestive system problems		1
				Good for the liver	1	
				Good for the stomach	2	5
				Vomiting		1
				Disinfectant	4	
			Tea with *Chelidonium*	Disinfectant	4	
			Tea	Hair care	4	
				Pain		1
				Panacea		1
				Aching legs		1
				Calming		2
				Toothache		3
				Cold	2	
*Acorus calamus* L. (Acoraceae) NB121	Аїр (Air)	Roots	Tea	Diarrhoea		1
				Good for the stomach		1
*Aesculus hippocastanum* L. (Sapindaceae) SB057 NB067	Castan; каштан; каштан кінський червоний; каштан чеворний (Kashtan; kashtan kinskyi chervonyi; kashtan chevornyi)	Flowers	Locally applied (in alcohol/moonshine)	Feet pain		1
				Joint pain	1	7
		Fruits	Infused in alcohol	Good for blood vessels		1
			Locally applied (in alcohol/moonshine)	Foot pain		1
				Joint pain		5
*Alchemilla vulgaris* auct. (coll.) (Rosaceae) SB039	Crețișoara; Гарник (Harnyk)	Aerial parts	Locally applied (infused in alcohol)	Joint pain	1	2
*Allium cepa* L. (Amaryllidaceae)	Ceapă; цибулька; цибуля (Tsybulka; tsybulia)	Bulbs	Raw	Blood pressure		1
				Iron		1
				Flu		2
			Tea	Fever	1	
				Cough	2	
				Good for the lungs	1	
			Boiled	Cough	1	3
			Raw (with honey and sugar)	Cough	1	
		External shell	Fomentation	Women’s problems		1
*Allium sativum* L. (Amaryllidaceae) NB192	Usturoi; часник (Chasnyk)	Bulbs	Raw	Blood cleansing		1
				Cancer		1
				Immune boosting		1
				Vitamins		1
			Raw	Flu		2
			Locally applied	Earache	2	
			Crushed and locally applied with massage	Flu		2
*Alnus glutinosa* (L.) Gaertn. (Betulaceae) NB050; NB052	Дубило§; вільха (Dubylo§; vilkha)	Bark	Boiled	Gangrene		2
*Aloe vera* (L.) Burm.f. (Xanthorrhoeaceae)	Алое (Aloe)	Leaves	Locally applied	Wounds		3
			Raw	Abscesses		1
*Anethum graveolens* L. (Apiaceae) SB032	Кріп (Krip)	Aerial parts, seeds	Tea	Panacea		1
		Leaves	Raw	Vitamins		1
		Seeds	Tea	Blood pressure		2
				Good for the stomach		2
				Fever		2
			Dried	To increase milk production in women		3
*Arctium lappa* L. (Asteraceae) SB052; SB091 NB149	Brusturi; Brusturoi; лопух; рипях; лопух; рипяка (Lopukh; rypiakh; rypiaka)	Flowers	Boiled	Hair care		2
		Leaves	Locally applied (fresh and crushed)	Joint pain	3	3
			Raw	Headache		2
		Roots	Boiled	Hair care		9
*Armoracia rusticana* P.Gaertn., B.Mey. & Scherb. (Brassicaceae) SB031 NB028; NB212	Hrean; хрін; хреню (Khrin; khreniu)	Leaves	Locally applied on the head (fresh)	Fever	1	
			Locally applied	Joint pain		1
		Roots	Raw (in food)	Help the bloodstream	1	
				Healthy	1	
			Locally applied	Joint pain		1
				Rheumatic pains		1
			Raw (in food)	Opening airways	1	
*Arnica montana* L. (Asteraceae)	Arnică; Arnic; Harnic; арніка (Arnica)	Flowers	Tea	Good for the heart		4
				Good for the eyes		1
			Locally applied (infused in alcohol)	Heart diseases		1
				Aching legs		2
				Back pain	1	
				Foot pain	2	
				Hand pain	2	
				Joint pain	4	8
				Rheumatic pains	5	1
				Wrist pain	1	
			Locally applied with (olive) oil	Hand pain	2	
				Joint pain		2
			Infused in alcohol (fresh)	Panacea	1^b^	
				Good for the skin	1^b^	
			Bath (fresh/dried)	Foot pain	1	
*Aronia melanocarpa* (Michx.) Elliott (Rosaceae)	горобина чорна; шкорух§; чорна горобина; рябина (Horobyna chorna; shkorukh§; chorna; horobyna; riabyna)	Fruits	Tea	Blood pressure		7
			Syrup	Blood pressure		1
*Artemisia absinthium* L. (Asteraceae) SB005 NB051	Pelin; полинь; полин (Polyn; polyn)	Aerial parts	Tea	Diarrhoea		1
				Good for the stomach	1	
				Stomach pain		1
			Tincture with alcohol	Appetite stimulant	2	
			Tea	Panacea		1
				Cancer		1^a^
		Seeds	Tea	Diarrhoea		1
*Atropa belladonna* L. (Solanaceae)	Матриган § (Matrygan)	Roots	Infused in alcohol/moonshine	Reproductive potency		1
				Cancer		1
				Good for women		1
				Joint pain		3
*Avena sativa* L. (Poaceae) NB202	Овес (Oves)	Seeds	Tea	Healthy		2
				Kidney stones		2
*Bellis perennis* L. (Asteraceae)	Маргаритки (Marharytky)	Flowers	Raw	Good for the heart		2
*Beta vulgaris* L. (Amaranthaceae) SB026	Sfeclă; буряк червоний (Buriak chervonyi)	Tubers	Any preparation	Anaemia	2	
			Juice	Blood cleansing		2
				Good for haemoglobin		2
				Healthy	1	
				Joint pain		2
				Headache		2
				Cough	1	2
				Good for the throat		2
*Betula pendula* Roth (Betulaceae) SB087 NB041; NB115	Mesteacăn; береза (Bereza)	Bark	Boiled	Gangrene		2
		Buds and leaves	Tea	Blood cleansing		1^b^
		Flowers	Tea	Good for kidneys		2
		Leaves	Boiled	Hair care		2
			Tea	Healthy		1
		Sap	Drink	Good for the heart	1	
				Vascular problems	1	
				Good for the stomach	1	
				Healthy	2	
				Good for the kidneys	2	1
				Good for the lungs	6	
				Lung cleansing	4	
		Young leaves	Compress	Joint pain		1
*Bidens tripartita* L. (Asteraceae) NB090	Череда (Chereda)	Aerial parts	Tea for bathing kids	Good for the skin		1
*Brassica oleracea* L. (Brassicaceae)	Varză; капуста (Kapusta)	Leaves	Fermented juice	Good for cholesterol		2
				Good for pancreas		2
				Good for the stomach		1
			Locally applied (fresh)	Frostbite	1	
			Poultice applied on the back	Fever	1	
			Locally applied (fresh)	Fracture	1	
				Joint pain		2
				Headache	1	2
*Bryophyllum pinnatum* (Lam.) Oken (Crassulaceae)	Каланхое (Kalancoe)	Sap	Drink	Rhinitis		2
*Calendula officinalis* L. (Asteraceae) NB233	Gălbenele; нагідки, крокіси§;крокіс§; календула; нагідки (Nahidky, krokisy ; krokis; kalendula; nahidky)	Flowers	Tea	Blood pressure		1
				Skin cleansing	1	
				Good for the liver	3	2
				Good for the stomach	2	
				100 diseases		2
				Immune boosting		2
				Inflammation processes		1
				Good for women		2
				Women’s problems		2
				Good for the kidneys		2
				Cough		1^b^
				Sore throat		1^b^
				Stomatitis (kids)		1^b^
			Boiled with fat and locally applied	Good for the skin	1	
				Warts	1	
				Fever	1	
			Syrup	Cough	1	
*Callisia fragrans* (Lindl.) Woodson (Commelinaceae)	золотий ус (zolotyi us)	Leaves	Tea	Blood cleansing		1^a^
*Cannabis sativa* L. (Cannabaceae)	Cânepă	Leaves	Burnt	Ear pain	1	
*Capsella bursa-pastoris* L. (Brassicaceae) NB218	Грицики (Hrytsyky)	Aerial parts	Tea	Blood pressure		1
				Women’s problems		1
				Headache		1
*Carum carvi* L. (Apiaceae) SB007 NB037	Săcărică; Secărică; Chimen; Hmel; Chimion; хміль§ (Khmil )	Aerial parts	Tea	Colds	3	
				Diarrhoea	5	2
				Good for the abdomen	2	
				100 diseases		2
				Healthy		2
				Strengthening of the organism	1	
				Cough	1	
				Good for the throat	1	
			Infused in alcohol	Hair care		1
		Seeds	Tea	Good for the stomach	5	5
*Centaurium erythraea* Rafn (Gentianaceae)	Центорія (Tsentoriia)	Aerial parts	Tea	Good for the heart		2
*Chelidonium majus* L. (Papaveraceae) SB003 NB154;NB078	Rostopască; чистотіл (Chystotil)	Aerial parts	Tea	Good for the digestive system	1	
				Good for the liver	2	
				Good for the stomach	2	
				Liver diseases	2	
				Organism cleansing	4	
				Stomach disinfection	4^a^	
			Locally applied (infused in alcohol)	Joint pain		1
			Tincture with vinegar	Joint pain		1^b^
		Sap	Locally applied (fresh)	Blisters	1	
*Chenopodium album* L. (Amaranthaceae) NB139	Натина§, лебеда (Natyna§;lebeda)	Aerial parts	Any Preparation	Healthy		1
*Cichorium intybus* L. (Asteracaeae) SB046	петрові батоги; петрів батіг (Petrovi batohy; petriv batih)	Aerial parts	Tea	Diarrhoea		1
				Good for the digestive system		1
				Good for the liver		1
*Coriandrum sativum* L. (Apiaceae)	коляндра; колєндра (Koliandra; koliendra)	Seeds	Tea	Fever		7
*Corylus avellana* L. (Betulaceae) SB089	Alune	Leaves	Tea	Prostatitis	2	
*Crataegus* spp*.* including *Crataegus monogyna* Jacq. (Rosaceae) SB064 NB066, NB234	Păducel; бояришнік; глід (Boiaryshnik^; hlid)	Flowers	Tincture with alcohol	Good for the heart		3
			Infused in moonshine/alcohol	Good for the heart		2
				Good for blood vessels		1
		Fruits	Tea	Blood pressure	1	2
				Good for cholesterol	1	
				Good for the heart	1	2
				Good for blood vessels		1
				Calming		1
				Soporific		1
			Dried	Good for the heart		1
			Tincture with alcohol	Good for the heart		3
*Cyanus segetum* Hill. (Asteraceae)	Centaurea; васильки (Vasylky)	Flowers	Tea	Panacea		1
		Aerial parts	Tea	Good for the liver	2	
*Daucus carota* L. (Apiaceae) SB027	Morcov	Roots	Raw	Improve vision	1	
*Dryopteris filix-mas* (L.) Schott (Dryopteridaceae) NB193	Лісова папороть; солодка папороть (Lisova paporot; solodka paporot)	Aerial parts	Boiled	Good for the heart		3
			Tea	Good for the heart		3
*Elaeagnus rhamnoides* (L.) A. Nelson (Elaeagnaceae)	Обліпиха (Oblipykha)	Fruits	Oil	Burns		1
				Wounds		1
			Raw with sugar	Healthy		1
			Boiled with sheep fat	Women’s problems		1
*Epilobium angustifolium* L. (Onagraceae) NB057	іван чай,демник§,димник§ (ivan chai,demnyk§, dymnyk§)	Flowers	Tea	Healthy		2
				Good for the intestines		1^a^
				Healthy		1^a^
*Equisetum arvense* L. (Equisetaceae) SB020 NB113;NB114	Coada calului; падиволос (хвощ) (padyvolos (khvoshch))	Aerial parts	Tea	Good for the abdomen	1	
				Liver diseases		1
				Good for the kidneys	1	
				Good for the urinary tract	4	
				Good for the lungs	2	
		Flowers	Infusion at 70°C	Headache	1	
*Fragaria vesca* L. (Rosaceae) SB094 NB004; NB015; NB071; NB240	Fragi; ягоди,лісова ягода; ягоди лісові; суниці лісові наз земляніка (yahody ; lisova yahoda; yahody lisovi; sunytsi lisovi naz zemlianika)	Aerial parts	Tea	Good for the heart	4	
				Healthy		1
		Flowers	Dried	Blood pressure		2
			Tea	Good for the kidneys		1
			Dried	Vitamins		3
			Dried	Diarrhoea		2
		Fruits	Raw	100 diseases		2
				Fever		1
				Healthy		1
				Good for the skin		2
*Frangula alnus* Mill. (Rhamnaceae)	Крушина (Krushyna)	Bark	Boiled	Jaundice		1
*Galium verum* L. (Rubiaceae) SB093	Sânziana	Aerial parts	Locally applied	Women’s problems	1^a^	
			Tea	Women’s problems	1^a^	
*Gentiana lutea* L. (Gentianaceae)	Gingiura; Джинджора (Dzhyndzhora)	Roots	Infused in alcohol	Good for the liver		1
				Good for the stomach	1	
*Ginkgo biloba* L. (Ginkgoaceae)	Гінго білоба (Hinho biloba)	Leaves	Infused in moonshine	Blood cleansing		1^a^
*Helianthus annuus* L. (Asteraceae)	Соняшник (Soniashnyk)	Fruits	Oil	Constipation		1
*Helichrysum arenarium* (L.) Moench (Asteraceae) NB258	Цмин пісковий (Tsmyn piskovyi)	Aerial parts	Tea (dried)	Good for the digestive system		1
				Stomach diseases		1
*Hypericum* spp. including *Hypericum perforatum* L. and , *Hypericum tetrapterum* Fr (Hypericaceae) SB068 NB080NB101; NB108; NB116	Pojărniță; Sunătoarea; звіробой; звіробій (Zviroboi; zvirobii)	Aerial parts	Tea	Blood pressure		2
				Blood cleansing		1^b^
				Diarrhoea		3
				Good for the liver	7	
				Good for the stomach	8	3
				Good for the gallbladder	1^a^	
				100 diseases		1
				Disinfectant	1	
				Healthy	1	4
				Panacea		1
				Women’s problems		2
				Calming	1	
				Good for the eyes	2	
			Drink	Evil eye		1^b^
			Locally applied (infused in oil)	Burns	4	1
				Wounds	4	
			Locally applied (in spirits with oil)	Good for the liver	2	
				Good for the stomach	2	
*Juglans regia* L. (Juglandaceae) SB051 NB210	Nuc; горіх (Horikh)	Flowers	Tea	Blood pressure		1
		Fruits	Dried	Healthy		3
				To increase milk production in women		2
			Raw	To increase milk production in women		3
				“Jod”		1
		Inner woody part of the fruit	Infused in alcohol	Good for the thyroid		1
		Leaves	Tea	Good for the heart		1
				Hair care	1	1^b^
		Unripe fruits	Infused in alcohol	Good for the thyroid		1
*Juniperus communis* L. (Cupressaceae) SB086	Ienupăr; жуніпера (zhunipera)	Fruits	Tea	Good for the liver	1	
		Leaves	Tea	Good for the kidneys		2
*Lamium album* L. (Lamiaceae) NB216	Кропива собача; біла кропива нежалка; глуха кропива (Kropyva sobacha; bila kropyva nezhalka; hlukha kropyva)	Aerial parts	Tea	Blood pressure		1
				Good for the heart		3
				Nerves		1
*Leonurus cardiaca* L. (Lamiaceae) SB013	Talpa gâștei; пустирник (Pustyrnyk)	Aerial parts	Tea	Blood pressure		1
				Good for the heart	6	1
				Healthy	2	
				Pain	1	
				Nerves	2	
				Rhinitis	4	
		Leaves	Locally applied (fresh with pork fat)	Cuts	2	
				Warts	2	
*Levisticum officinale* W.D.J.Koch (Apiaceae)	Любисток (Liubystok)	Aerial parts	Tea	Alcoholism		1
				Hair care		3
*Lilium candidum* L. (Amaryllidaceae) SB049	Crin alb; Narcise; лілія біла; лилия (Liliia bila; lylyia)	Flowers	Locally applied (infused in alcohol) Locally applied (infused in alcohol) Locally applied (in spirits, medicinal)	Good for veins	1	
				Bee stings		1
				Burns		1
				Warts		5
				Wounds		6
				Joint pain	1	
				Tired feet	1	
			Drink (infused in alcohol)	Healthy		1
*Linum usitatissimum* L. (Linaceae)	Lin; лен; льон (Len; lon)	Seeds	Tea	Good for the stomach	1	2^b^
				To increase milk production in women		3
*Lonicera caprifolium* L. (Caprifoliaceae)	Floarea maicii domnului	Aerial parts	Locally applied (dried tea)	Wounds	1	
				Women’s problems	1	
				Measles	1	
*Lycopodium clavatum* L. (Lycopodiaceae) NB231	Плаун (Plaun)	Aerial parts	Dried	Wounds		2
*Maclura pomifera* (Raf.) C.K.Schneid. (Moraceae)	Адамове яблуко матлюрка (Adamove yabluko matliurka)	Fruits	Locally applied (infused in alcohol)	Women’s problems		2
				Joint pain		2
*Malus domestica* Borkh. (Rosaceae) NB242	Яблука (Yabluka)	Fruits	Boiled with onion	Cough		3
*Matricaria chamomilla* L. (Asteraceae) SB019; SB022 NB164;NB171	Mușețel; Romaniță; ромашка; румянець (Romashka; rumianets)	Aerial parts	Tea (dried)	Red skin		1
				Good for the digestive system		1
				Inflammation processes		1
				Good for the throat		1
		Flowers	Poultice (dried)	Evil eye	1	
			Compress	Skin infections	1	
				Warts	1	
				Eye cleaning	2	
				Eye problems	1	3
				Good for the eyes	1	2
			Tea	Diarrhoea	1	
				Good for the stomach	2	3
				Disinfectant	1	
				Healthy	1	3
				Panacea	1	5
				Good for the urinary tract	2	
				Headache	2	
				Wound cleansing		1^b^
				Gum problems	1	
				Colds	2	1^c^
			Tea with *O. Vulgare*	Gum problems	1	
				Disinfectant	1	
*Melissa officinalis* L. (Lamiaceae)	Меліса (Melisa)	Leaves	Tea (dried)	Healthy		2
				Pain		2
				Calming		3
				Headache		1
				Soporific		1
				Stress		1
*Mentha* spp. (Lamiaceae) SB014; SB016; SB034; SB096 NB079;NB080;NB097	Mentă; мята; мятка; мята гладка; мята кучерява; мятка кінська; мятка перчева (Miata; miatka; miata hladka; miata kucheriava; miatka kinska; miatka percheva)	Aerial parts	Tea	Good for the heart		6
				Heart disease		1
				Diarrhoea	1	
				Good for the stomach		1
				Stomach problems		2
				Vomiting		1
				Healthy	1	
				Pain		1
				Diuretic	1	
				Good for the urinary tract	2	
				Calming	1	1
				Headache		2
				Stress		1
			Locally applied (infused in alcohol)	Joint pain		1
*Origanum vulgare* L. (Lamiaceae) SB036 NB033; NB055; NB021	Șovârv; Șovârf; Materanca bila; материнка (Materynka)	Aerial parts	Tea	Blood pressure	4	
				Blood regeneration		1
				Good for the heart	4	2
				Red skin		2
				Abdominal pain	2	
				Diarrhoea		1
				Good for the liver	3	
				Good for the stomach	7	2
				100 diseases		2
				Disinfectant	1	
				Fever	1	
				Healthy		2
				Inflammation processes		2
				Panacea		1
				Leptospirosis	1	
				Septicaemia	1	
				Good for the kidneys	1	
				Soporific		1^b^
				Good for the lungs		1
		Aerial parts (flowers)	Tea	Women’s problems		8
*Panax ginseng* C.A. Mey. (Araliaceae)	Женшень (Zhenshen)	Roots	Infused in alcohol	Blood pressure		1
*Papaver somniferum* L. (Papaveraceae)	Мак (Mak)	Aerial parts	Tea	Soporific		2^b^
*Petroselinum crispum* (Mill.) Fuss (Apiaceae) NB220	Петрушка (Petrushka)	Leaves	Raw	Vitamins		2
*Phaseolus vulgaris* L. (Leguminosae)	Фасоля (Fasolia)	Pod	Tea	Diabetes		2
*Picea abies* (L.) H. Karst. possibly including *Abies alba* Mill. (Pinaceae) SB008 NB043	Brad; смерека; хвоя (Smereka, khvoya)	Flowers	Syrup	Bronchitis		2
				Cough		4
				Good for breathing		2
		Needles	Syrup	Cough		2
			Tea	Cough		2
				Good for the throat		2
		Resin	Locally applied	Joint pain		2
		Sprouts (young)	Syrup	Fever	1	
				Panacea	2	
				Colds	4	
				Cough	8	
				Good for the lungs	2	
				Good for the respiratory system	2	
				Good for the throat	1	
				Bronchitis		2
				Sore throat	1	
			Essence (fresh)	Panacea	2	
				Colds	2	
		Young cones	Syrup	Bronchitis		2
				Cough		4
			With sugar	Pneumonia		1
*Pinus sylvestris* L. (Pinaceae)	Pin	Young sprouts	Syrup (fresh)	Cough	1	
*Plantago lanceolata* L. (Plantaginaceae) SB037	Pătlagină îngusta; подорожник ланцеолистий (Podorozhnyk lantseolystyi)	Leaves	Tea (fresh)	Cough	1	1
*Plantago major* L. (Plantaginaceae) SB066 NB022;NB047; NB132	Platagine; Platagină; Podorojnic; подорожник (Podorozhnyk)	Leaves	Locally applied (fresh)	Abscesses		2
				Cuts	1	1
				Good for the skin	2	
				Pus	1	
				Skin infections	1	
				Skin irritation	1	
				Disinfectant		1^b^
				Sores	1	
				Warts	3	3
				Wounds	1	15
			Locally applied (with alcohol)	Wounds		2
			Tea	Cough		2
		Seeds	Tea	Good for the kidneys		2
		Whole plant	Tea	Women’s problems		1
*Potentilla anserina* L. (Rosaceae)	Coada racului	Aerial parts	Tea	Indigestion	1	
*Potentilla erecta* (L.) Raeusch. (Rosaceae)	Калган; калган; перстач прямостоячий (Kalhan; kalhan; perstach priamostoiachyi)	Roots	Tea	Reproductive potency		1
			Tea (dried)	Reproductive potency		1
			Infused in alcohol	Good for men		1
				Joint pain		2
				Good for the thyroid		1
*Primula* spp. including *Primula veris* L. and *Primula elatior* (L.) Hill (Primulaceae)	Ciobațica cucului; Cioboțica cucului; первоцвіт буковинський (Pervotsvit bukovynskyi)	Aerial parts	Tea	Good for the heart	1	
				Good for the liver	1	
				Good for the stomach		2
		Flowers	Tea	Cough		3
*Prunus avium* (L.) L. (Rosaceae) SB059	Cireș	Stalks	Tea	Diuretic	1	
				Good for the kidneys	1	
*Pteridium aquilinum* (L.) Kuhn (Dennstaedtiaceae) NB074	Папороть орляк (Paporot orliak)	Aerial parts	Bath	Women’s problems		1
*Pyrus pyraster* (L.) Burgsd*.* (Rosaceae)	Дика груша (Dyka hrusha)	Fruits	Tea	Salt in the joints		2
			Infused in spirits	Salt in the joints		2
*Quercus* spp. including *Quercus robur* L*.* and *Quercus rubra* L.	Дуб (Dub)	Bark	Boiled	Gangrene		2
			Tea	Toothache		1
*Raphanus sativus* L. (Brassicaceae) NB250	Редька чорна (Redka chorna)	Roots	Baked	Cough		2
				Good for breathing		2
*Ribes nigrum* L. (Grossulariaceae) SB042 NB211	Coacăză neagră ; смородина; чорна смородина (Smorodyna; chorna smorodyna)	Aerial parts	Tea	Cough		2
		Fruits	Juice	Blood pressure		1
			Jam	Good for haemoglobin		1
			Jam	Good for the eyes		1
			Raw	Blood pressure		3
				Good for the intestines	2	
				Vitamins	2	
*Ribes rubrum* L. (Grossulariaceae) SB042 NB213	Coacăză rosu; яверниці§; пожички§;червона смородина (Yavernytsi§, pozhychky§, chervona smorodyna)	Fruits	Raw	Good for the intestines	2	
				Vitamins	2	
				Kidneys stones		2
			Tea	Fever		1
				Flu		1
*Rosa canina* L. (Rosaceae) SB062 NB018; NB083	Măceșe; Măceș de padure; шипшина (Shypshyna)	Fruits	Tea	Good for the heart	1	
				Good for the kidneys		2
				Healthy		2
				Immune boosting		1
				Vitamins		1
				Good for the kidneys		1
				Good for the urinary tract	2	
				Cough	4	
				Flu	4	
			Syrup (fresh)	Cold	1	
		Roots	Tea	Good for the kidneys		3
*Rosa rugosa* L. and *Rosa centifolia* L. (Rosaceae) SB023	Trandafir; роза (Roza)	Flowers	Tea	Calming		1
		Petals	Juice	Foot-and-mouth disease	1	
*Rubus caesius* L. (Rosaceae) NB062; NB063	Ожина; чониця; єжевіка; ожина (Ozhyna; chonytsia; yezhevika; ozhyna)	Fruits	Infused in alcohol	101 diseases		2
			Boiled	Healthy		1
			Raw (with sugar)	Cancer		1
				Vitamins		5
				Colds		1
				Flu		1
*Rubus idaeus* L. (Rosaceae) SB071; SB009 NB081	Zmeură; малина (malyna)	Aerial parts	Tea	Diabetes	1	
				Diabetes		1
				Fever	3	6
				Healthy		3
				Good for the kidneys		1
				Colds	2	3
				Cough		3
				Flu		1
		Fruits	Tea	Blood pressure		2
			Jam	Good for haemoglobin		1
			Dried	Fever		3
			Juice	Fever		6
				Colds	1	
				Cough	1	
			Juice With Lemon	Fever	1	
			Locally applied syrup without sugar	Fever	1	1
			Moonshine	Healthy		1^a^
			Syrup (without sugar, with mashed potatoes )	Fever	1	3
				Colds	1	
			Raw (with sugar)	Panacea		1
				Flu		1
				Vitamins		5
				Colds	2	1
				100 diseases		2
			Syrup (fresh)	Cough	1	
				Flu	4	1
				Colds	1	
				Fever	5	
				Strengthening of the organism	1	
				Good for the lungs	1	
*Rumex acetosa* L. (Polygonaceae) NB081	Măcriș; квас§; квасок§; щавель (Kvas§; kvasok§; shchavel)	Aerial parts	Any preparation	Vitamins		3
		Roots	Tea	Good for the liver	1	
				Colds	2	
*Rumex alpinus* L. (Polygonaceae) SB067	Ștevie	Leaves	Locally applied (fresh)	Cuts	2	
			Tea	Diarrhoea	1	
*Salix × fragilis* L. (Salicaceae)	Верба ламка (Verba lamka)	Bark	Tea	Fever		1
*Salvia pratensis* L. (Lamiaceae) SB028	Salvia	Aerial parts	Tea	Calming	1	
*Sambucus nigra* L. (Adoxaceae) SB084 NB054	Soc; бузина (Buzyna)	Flowers	Drink	Blood pressure	1	
				Good for the stomach	4	
				Good for the kidneys	4	
				Good for the urinary tract	4	
			Tea	Colds		1
				Cough		4
				Flu		2
				Good for the throat		2
			Infused in alcohol	Cough		1
		Leaves	Dried	Good for the heart		2
*Sedum roseum* (L.) Scop. (Crassulaceae)	Червона щітка (Chervona shchitka)	Roots	Tea	Good for the pancreas		1
				Immune boosting		1
*Solanum tuberosum* L. (Solanaceae)	Cartofi; бараболя; картошка (Barabolia; kartoshka)	Tubers	Locally applied (fresh)	Fever	1^b^	3
				Headache	3	
			Boiled	Cough		3
*Sorbus domestica* L. (Rosaceae) SB055 NB232	Scoruș	Fruits	Tea (fresh/dried)	Blood sugar	4	
*Stellaria media* (L.) Vill. (Caryophyllaceae)	Мокриця; червець (Mokrytsia; chervets)	Aerial parts	Tea	Cough		2
*Symphytum officinale* L. (Boraginaceae) SB070 NB166; NB167; NB184; NB189	Tătăneasă; живокост; гауізь§; гауїзь§ (Zhyvokost; hauiz§; hauiz§)	Roots	Locally Applied (boiled)	Good for the skin	1	
			Locally applied (fresh)	Fracture	1	1
				Gout		1
				Joint pain	1	3
				Rheumatic pains		1
				Hernia	1	
			Locally applied (infused in alcohol)	Joint pain	2	
				Good for the liver	2	
			Locally applied (fresh with wax)	Joint pain		1
		Whole plant	Tea (fresh)	Good for the liver	2	
				Good for the stomach	2	
*Syringa vulgaris* L. (Oleaceae) NB208; NB209	Бузок (Busok)	Flowers	Infused with moonshine	Joint pain		1
			Tea	Bronchitis		1
				Cough		3
*Tagetes erecta* L. (Asteraceae)	Чорнобривці (Chornobryvtsi)	Flowers	Tea	Blood cleansing		1
				Abscesses		1
				Diabetes		1
				Good for the liver		1
*Tanacetum balsamita* L. (Asteraceae)	Canufar; Кануфер (Kanufer)	Aerial parts	Infused in alcohol	Abscesses		1
				Wounds	1^b^	1
*Taraxacum officinale* Webb (Asteraceae) SB063 NB016;NB048	Păpădie ; кульбаба (Kulbaba)	Aerial parts	Raw	Vitamins		2
		Flowers	Syrup (fresh)	Good for the liver	1	
			Jam	Good for the urinary tract	4	
*Thymus* spp. including *Thymus serpyllum* L. and *Thymus vulgaris* L. (Lamiaceae) SB001; SB090 NB030; NB027; NB125; NB019	чабер; чебрець, чебрик; городній чебрець (Chaber; chebrets, chebryk; horodnii chebrets) *Thymus serpyllum*: Cimbrișor; чебрець звичайний, чебрик польовий; польовий чебрець (chebrek polovyi; chebrets zvychainyi;chebryk polovyi; polovyi chebrets) *Thymus vulgaris*: Cimbru sălbatic; чеберець садовий (Cheberets sadovyi)	Aerial parts	Tea	Good for the stomach	1	2
				Lung diseases		1
				Good for the throat		1
				Good for the lungs	3	
				Colds	2	
				Pain	2	
				Panacea	3	
				Good for the kidneys	1	
			Syrup	Cough		3
			Tea	Good for breathing		1^b^
				Cough	10	12
				Good for veins	2	
				Alcoholism		1
			Burnt three times	Evil Eye		1^b^
		Flowers	Tea	Colds		1
*Tilia cordata* Mill. (Malvaceae) SB017 NB253	Tei; липа (Lypa)	Flowers	Tea (dried)	Good for the heart	4	
				Abdominal pain	1	
				Good for digestion	2	
				Good for the liver		2
				Good for the stomach	7	
				Fever		2
				Inflammation processes		1
				Organism cleansing	1	
				Good for women	1	
				Good for the kidneys		2
				Calming	6	1^b^
				Headache	1	
				Headache	1	1
				Soporific	2	
				Colds	2	1
				Cough	3	1
				Flu	1	
				Panacea		1^b^
		Leaves	Boiled	Hair care		2
*Trifolium pannonicum* Jacq. (Leguminosae)	конюшина панойська з жовтими квітами (Koniushyna panoiska z zhovtymy kvitamy)	Flowers	Tea (dried)	Healthy		1
*Trifolium sp*. including *Trifolium pratense* L. (Leguminosae) SB072; SB075; SB077; SB078 NB002; NB013; NB014; NB076; NB086; NB102; NB103; NB110; NB111; NB112; NB119; NB123; NB126; NB134; NB140; NB144	Trifoi alb; Trifoi rosu; тріфоль; конюшина червона (Trifol; koniushyna chervona)	Aerial parts	Tea (dried)	Good for the urinary tract	2	
				Headache	1	1
				Good for the lungs	4	
*Tussilago farfara* L. (Asteraceae) SB065; SB085 NB072; NB133	Podbal; підбіл;мати й мачуха; (Pidbil; maty y machukha)	Aerial parts	Tea	Cough	1	9
		Flowers	Syrup (fresh)	Cough	1	
			Tea	Colds		1
		Leaves	Locally applied (fresh)	warts	2	
		Roots	Syrup	Good for the throat		1
		Whole plant	Boiled	Cough		1
*Urtica dioica* L. (Urticaceae) SB088 NB026; NB048	Urzică; кропива; кропива жалка (Kropyva; kropyva zhalka)	Young sprouts (aerial parts)	Boiled (in soup)	Blood cleansing	4	1
				Vessel cleansing		1
			Tea	Blood cleansing	9	3
				Blood pressure		1
				Good for the heart	1	2
				Good for the stomach		2
				Vomiting		2
				Rheumatic pains	2	
				Calming		2
				Toothache		2
				Nosebleeds		2
				Healthy	1	
				Organism cleansing	5	
			Boiled	Hair care	5	10
			Any preparation	Healthy		1
				Panacea	2	
				Vitamins	2	3
			Locally applied (fresh)	Rheumatic pains	4	
*Vaccinium myrtillus* L. (Ericaceae) SB006 NB060	Afina (fruits); Afiniș (aerial parts); афини; чорниця (fruits); аффинник (aerial parts) (Afyny; chornytsia; afynnyk)	Aerial parts	Tea	Blood cleansing	1	
				Blood pressure		2
				Good for the heart	1	
				Fever	1	
				Healthy	1	
				To be strong		1
				Lowering glycaemia	1	
				Good for the eyes		3
			Compress	Diabetes	2	
			Any preparation	Diabetes	6	1
				Good for the stomach	11	7
			Compress	Eye problems	2	1
		Aerial parts ( including fruits)	Tea	Good for the kidneys	1	4
		Flowers	Dried	Good for the pancreas		1
				Good for the stomach		1
		Fruits	Raw (with sugar)	Good for the heart	2	
				Diabetes		1
				Diarrhoea		1
				Healthy	2	5
				Panacea	3	1
				Improve vision	2	
				Vitamins		2
				Good for the eyes		4
				Flu		1
			Jam	Flu		1
				Good for the eyes		2
				Good for haemoglobin		1
				Diarrhoea		1
			Juice	Diarrhoea	1	
				Good for the liver	1	
			Syrup	Abdominal pain	1	
				Good for the abdomen	1	
				Diarrhoea	1	3
				Appetite suppressant	1	
				Panacea		2
				Good for the liver	2	
			Infused raw in alcohol	Good for the stomach		4
				Stomach diseases		2
				100 diseases		6
				Good for the eyes		2
				Stomach pain		2
			Dried Dried	100 diseases		2
				Good for the eyes		2
			Tea	Panacea		2
				Good for the eyes		3
				Diarrhoea		6
				Improve vision	1	
			Tincture	Flu		1
*Vaccinium vitis-idaea* L. (Ericaceae) SB010 NB061	Merișoare; Gogoze§; брусниця; ґоґодзи§; гогдзі§ (Brusnycia, gogodzy§; hohdzi§)	Aerial parts	Tea	Good for the heart	1	
				Diabetes		1
				Diarrhoea	1	
				Good for the liver	1	
				Good for the stomach	1	
				Fever	1	
				Good for the kidneys	1	5
				Kidney diseases	1	
				Urinary tract diseases	1	
		Fruits	Any preparation	Blood pressure		3
			Raw	Blood pressure		1
				Panacea		1
				Vitamins		4
				Good for the kidneys		3
				Urolithiasis		1
				Good for the heart	1	
			Tea	Blood pressure	1	
				Panacea		2
				Good for the heart	1	1
			Compote	Fever		1
			Juice	Fever	1	
			Syrup (without sugar)	Fever	1	
			Water source and fresh fruits/ compote	Immune boosting	1	1
			Compote	Cough		1
			Any preparation	Flu		1
		Roots	Tea	Good for the bladder		1
*Valeriana officinalis* L. (Caprifoliaceae)	Валеріана (Valeriana)	Roots	Infused in alcohol	Heart disease		1
*Viburnum opulus* L. (Adoxaceae) NB223	Călină; калина (Kalyna)	Flowers	Tea	Fever		2
		Fruits	Tea	Blood pressure		7
				Good for the heart	4	
				Panacea	2	
				Cold	2	
				Cough	4	
				Good for the lungs	4	
				Fever		2
			Syrup	Blood pressure		2
				Flu		1
				Fever		1
			Raw (with sugar)	Good for the heart		1
				Panacea		1
		Leaves	Boiled	Joint pain		1
*Vitis vinifera* L. (Vitaceae) NB204	Виноград (Vynohrad)	Fruits	Wine	Good for blood		1
				Panacea		1

Plant names mentioned by Ukrainian Hutsuls are reported in Cyrillic (with transliteration). Plant names mentioned by Romanian Hutsuls are reported in the Latin alphabet. Plant names not reported in Romanian or Ukrainian dictionaries or in previous publications (e.g. Pieroni and Soukand, 2017), and are therefore probably Hutsul names, are marked with a §. A Russian name is marked with a ^

^a^denotes a recently adopted use

^b^denotes a past use

The most common medicinal taxon was the same in both communities, namely *Vaccinium myrtillus* (78 DUR among Ukrainian Hutsuls and 45 DUR among Romanian Hutsuls). In Northern Bukovina, it was followed by *Rubus idaeus* (46 DUR), *Urtica dioica* (32 DUR), *Plantago major* (31 DUR) and *Vaccinium vitis-idaea* (27 DUR). In Southern Bukovina, it was followed by *Urtica dioica* (35 DUR), *Hypericum* spp. (33 DUR), *Tilia* spp. (32 DUR) and *Rubus idaeus* (27 DUR). Half of the reported medicinal DURs on both sides of the border are for cultivated plants, while wild species represent 24% and 31% of the reported taxa in Northern and Southern Bukovina, respectively.

**Fig. 6:**
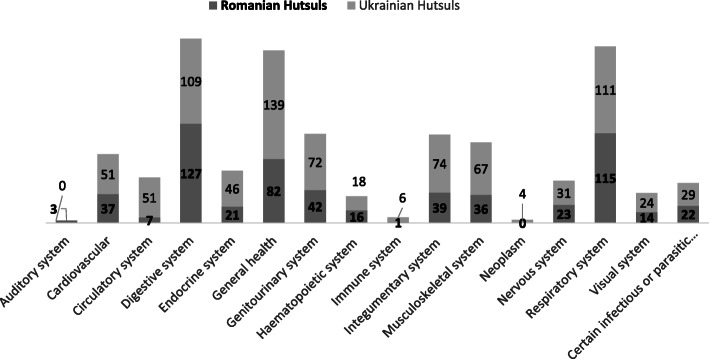
The distribution of medicinal DUR for the ICD-11 system categories shows that general health was the most important category among Ukrainian Hutsuls, while the digestive system was the most reported among Romanian Hutsuls. Both groups reported treating disorders of the respiratory system with medicinal plant preparations

Romanian Hutsuls particularly mentioned medicinal taxa for treating the respiratory system, the digestive system and for general health (Fig. 6). In the first two cases, they reported more DURs than did Ukrainian Hutsuls. In Northern Bukovina, the first three medicinal categories reported by Hutsul interviewees were general health, the respiratory system and the digestive system.

Following the higher number of medicinal DUR among Ukrainian Hutsuls, they exceeded Romanian Hutsuls in all medicinal categories apart from those of the auditory, digestive and respiratory systems. Interestingly, cancer (neoplasm category, four taxa) was only mentioned in Ukraine, whereas in Romania two people reported a remedy for cancer using *Helleborus foetidus*, but then added that they do not to use it as it is very dangerous.

We recorded a total of 128 emic medicinal categories which were nearly equally distributed: 42 were reported by both communities, 41 among Romanian Hutsuls and 45 among Ukrainian Hutsuls.

Only ten medicinal DURs used by at least 10% of each community were found on both sides of the border. Three DURs were included in the digestive category and specifically considered as good for the stomach: tea made from the seeds of *Carum carvi* (used by one fifth of the interviewees), dried aerial parts of *Hypericum perforatum* and any preparation of *Vaccinium myrtillus*. Two musculoskeletal remedies include compresses of the leaves of *Arctium lappa* and the flowers of *Arnica montana* infused in alcohol, locally applied to treat joint pain. The aerial parts of *Rubus idaeus* are prepared as tea to reduce fever, while the aerial parts of *Urtica dioica* are boiled and used to wash the hair (for strong and shiny hair). More than 30% of both communities consider *Thymus* spp. as a remedy for cough. Finally, the fresh leaves of *Plantago major* are locally applied to warts and the young sprouts of *Urtica dioica* are considered beneficial for cleansing the blood.

### Knowledge transmission

**Fig. 7:**
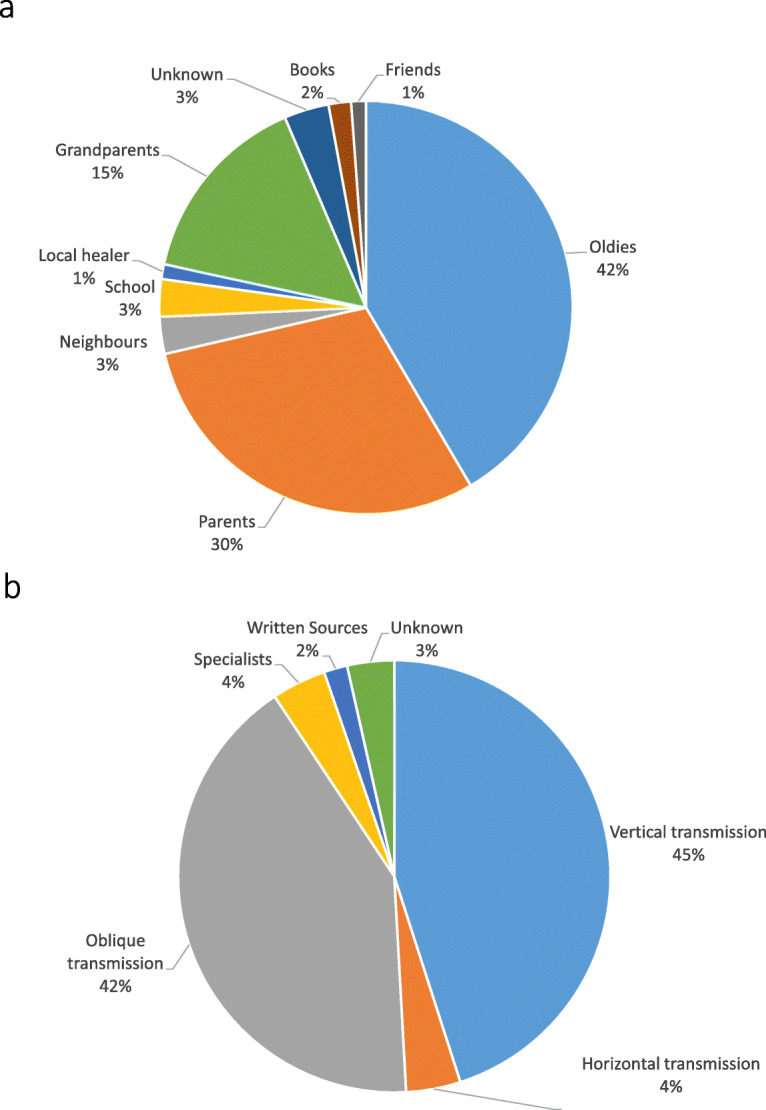
**a** Ecological knowledge transmission strategies among Romanian Hutsuls. **b** Ecological knowledge transmission categories among Romanian Hutsuls grouped per strategy

We recorded eight sources of knowledge among both Romanian and Ukrainian Hutsuls. Three categories differ between the two groups: friends, professors and a local healer (in the past) were mentioned in Southern Bukovina, while television, the Internet and newspapers were mentioned in Northern Bukovina. When analysing these data in the framework of the abovementioned Van den Boog [10] study, we observed that in 45% of cases Romanian Hutsuls transferred their knowledge vertically (from parents, grandparents and great-grandparents), 42% obliquely (via the elderly of the village) and 4% horizontally (through friends and neighbours), while 4% received knowledge from specialists (local healers and professors) and written sources (books) accounted for 2% (Fig. 7). Among the books, one elderly interviewee mentioned Maria Treben’s [29] bestseller (for the preparation of *Primula* tea), but most of the Romanian Hutsuls said they did not have time for reading as there was always a lot of work in maintaining their small-scale farms. Moreover, all the Romanian people who mentioned books as a source of knowledge added that they would never have trusted this information as such, but they had a solid base of knowledge derived from oral sources and they have just added some information to it (for instance, they did not know a specific plant was useful for something, but they were already using it or part of it).

**Fig. 8:**
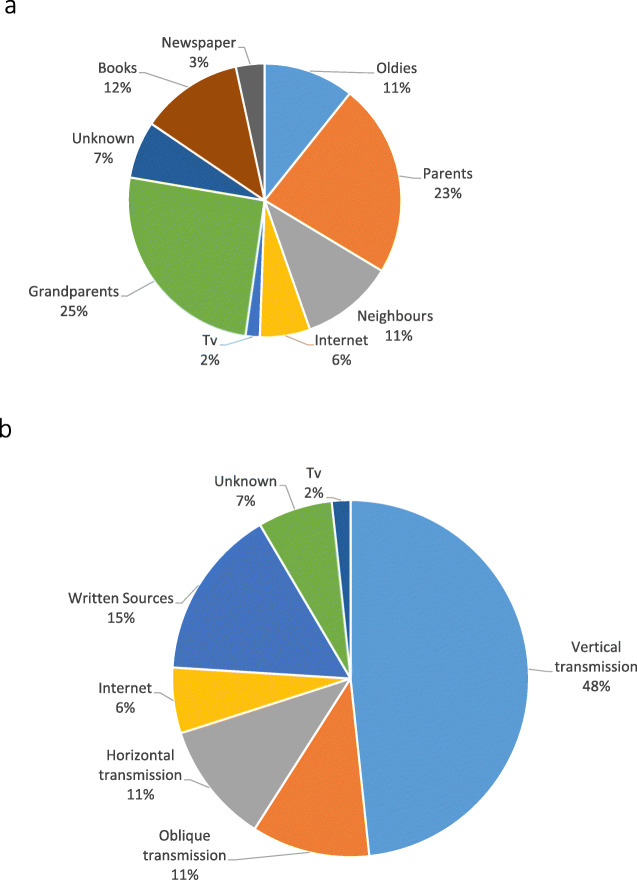
**a** Ecological knowledge transmission strategies among Ukrainian Hutsuls. **b** Ecological knowledge transmission strategies among Ukrainian Hutsuls grouped per strategy

Among the Ukrainian Hutsuls, we recorded nearly the same proportion of vertical ecological knowledge transmission from parents and grandparents (48%), as well as the same amount of horizontal transmission from neighbours and oblique transmission from local elderly individuals (11%) (Fig. 8). We also observed that 15% of knowledge was obtained from written sources including books and newspapers (‘I read in the newspaper that a bath with *Chelidonium majus* and *Matricaria chamomilla* helps with allergies’, explained a women born in 1969), 6% from the Internet and 2% from television.

Comparing the two communities, we can observe that the elderly, and thus oblique LEK transmission, play a minor role among Ukrainian Hutsuls, while neighbours have a more important role (‘Come to my neighbour, she knows everything’, advised an old woman born in 1928). Also, in Ukraine, no one reported having learnt from specialists, while mass media such as the Internet and television accounted for 8%, which added to the 15% from books and newspapers totals 23%, whereas this value is only 12% among Romanians.

Moreover, while only one book was mentioned [28] and another one was presented during interviews in Southern Bukovina [30], Ukrainian Hutsuls reported 16 books in both Ukrainian and Russian published between 1979 and 2016 (Table 3).

**Table 3:** Details of the books reported during interviews in Northern Bukovina, Ukraine

Author	Year	Title	Publisher/City	Language	No of pages
Alekseev I. Dibrova A.	2012	Complete atlas of medicinal plants	Gloria, Kiev	Russian	400
Grechanyi I.	2015	The Great Illustrated Directory of Medicinal Herbs	Book club ‘Family Leisure Club’, Kharkiv	Ukrainian	544
Grodzinsky AM.	1990	Medicinal plants: Encyclopedic reference book	‘Ukrainska encuklopedia’ MP Bazhana, Kiev	Ukrainian	544
Markova A.	2002	The Complete Encyclopedia of Folk Medicine	Esmo, Moscow	Russian	640
Pavlenko L.	1992	Drugs from Chardzilla	Veselka, Kyiv	Ukrainian	52
Reutov S.	2016	Natural healers of 1000 diseases	Book club ‘Family Leisure Club’, Kharkiv	Russian	320
Rosola T. Rosola I. Rubish F.	2012	Medicinal plants of Transcarpathia in folk medicine	Patent, Uzgorod	Ukrainian	208
Ivashyn D. Katina Z. Rybachuk I. et al.	1983	Directory of preparations of medicinal plants harvest	Urozai, Kev	Russian	296
Safonov MM.	2015	Full atlas of medicinal plants	Bogdan, Ternopil’	Ukrainian	384
Schultz J. Uberguber E.	1994	Medicines from God's Pharmacy	Anfas, Kiev	Russian	207
Smik GK.	1991	Useful and rare plants of Ukraine	Ukrainska radyanska encuklopedia, Kiev	Ukrainian	416
Smolinskaya M. Korolyuk V. Galitska L.	2002	Medicinal plants of Bukovina	Ruta, Cernivtci	Ukrainian	295
Sokolov C. Zamotayev I.	1988	Directory of Medicinal Plants	Nedra, Moskow	Russian	464
Uzhegov H.	2011	The Complete Encyclopedia of Folk Medicine	Astrel, Moskow	Russian	1088
Henzel W.	2016	An illustrated herbalist. 350 species	Family Leisure Club, Kharkiv	Russian	256
Yelin Y.	1979	Plants of our forests	Soviet School, Kiev	Ukrainian	239
Zinchenko TV. Stakhiv IV. Myakushko T.	1990	Medicinal plants in gastroenterology	Naukova Dumka, Kiev	Russian	240

#### Popular books about medicinal plants in Northern Bukovina (Ukraine)

Books on medicinal plants were very popular in Ukraine and could be grouped based on the period of their publication. The first period of mass publication of books on wild medicine began in the 1970s. At that time, most of the books had an official reviewer controlled by Moscow, as a rule a doctor or professor of medicine. The popularity of herbal medicinal books can be seen by the number of editions; for example, Dr. Karhut’s ‘Medicine around us’ was republished in 1975, 1978 and 1979. Hammerman and co-authors published the text book ‘Medical plants or plant-helpers’ in 1978 and then again in 1979 for biological specialties and medical schools, which was adopted by the Ministry of Education of the USSR.

The second period started at the beginning of the 1990s when there were no longer censors, and therefore a boom of book publications took place; and indeed out of the 16 books mentioned during our interviews, 11 are from this period. Besides books, respondents named a variety of newspapers that specialized in recipes of wild and domesticated taxa for medicinal purposes. We recorded eight different newspapers and magazines named by interviewees, e.g. ‘Alphabet of health’, ‘Health advice’, ‘Good doctor’ and ‘Granny’. These magazines were very cheap and promoted by the state postal service. Those publications included recipes from medical doctors as well as from people that ‘treated themselves’ with specific remedies.

#### Different attitudes towards written and visual sources among Hutsuls on the two sides of the border

We observed a different attitude towards written sources between the two communities. While in Romania books were somehow perceived as unnecessary, not completely useful (as *the elderly know more*) and not to be trusted (as *the elderly know better*), in Ukraine they were a real source of pride. ‘We are very knowledgeable people, we go to libraries’, claimed a woman (born in 1966). Indeed, in Ukraine during the Soviet era, education and books were important ways of showing off, as boasted by a Hutsul woman (born in 1948): ‘I have an expensive book! (the medicinal plants book)’. This is because books were very rare and hard to get during Soviet times [31]. Therefore, the large number of books shown during our interviews may be due to informants’ pride of being able to show that they are knowledgeable people who have the economic power to buy books and can acquire ‘high’ knowledge (compared to the lesser importance of oral knowledge). Specifically, books regarding medicinal uses of plants were propagandized and it was a popular topic in schools and universities. In addition, phytotherapeutic knowledge was especially sought-after because the Soviet medical system relied heavily on herbal medicine, e.g. a special course on herbal medicine was offered at all medical universities of Soviet Ukraine [32,3 3]. Indeed, this positive attitude towards ‘official’ and written sources has been observed in other post-Soviet countries and confirms that book knowledge is considered especially trustworthy in these contexts [32].

Another difference between Ukrainian and Romanian Hutsuls is that neighbours are an important source of knowledge among the former, while a similar role is played by the elderly among the latter. Although it may simply be a phenomenon related to semantics (elderly individuals can also be neighbours), there may be more older and knowledgeable people in Romania, as in Ukraine a particular generation was deported to Siberia and never returned, or if individuals did return they did not live long [33] or were killed during WWII and the time of repressions [34].

### Different perspectives on Hutsul ecological knowledge transmission patterns on both sides of the border

Our overall data reveals that LEK among Romanian and Ukrainian Hutsuls is transferred using different transmission patterns and sources. Indeed, among Romanians, the main rule seemed to be the experiential ‘uite, asta-i buna sau nu-i buna (look, this is good, this is not)’ learnt from parents or the elderly of the village, as an 85-year-old Hutsul man reported. This attitude is clearly encompassed by the definition of traditional knowledge, as it is transmitted orally in the local language and characterized by ubiquitous dissemination. The other sources of knowledge accounted for only 6% in total.

Among Ukrainian Hutsuls, there is a larger proportion of knowledge that comes from other sources (23%). However, even though magazines and pamphlets were found to be an important source of knowledge in several post-Soviet countries [35, 36], the Internet and television were not found to influence medicinal plant knowledge in other areas of Ukraine [37].

#### Socio-political factors affecting LEK in Northern Bukovina (Ukraine)

The reasons for the different knowledge transmission strategies may be found in the distinct social and political environments which the ‘new’ border created. In Northern Bukovina, Hutsuls were part of a centripetal system that delivered services and information equally to every part of the USSR.

The educational system promoted by the Soviet Union significantly impacted the Hutsul way of thinking and living [38]. All across Ukraine, this was implemented through both the mandatory teaching of the Russian language, which was required for any prestigious job [39], and the promotion of ‘rural clubs’, which proposed new forms of political education such as mobile libraries and cinemas in order to reach people in even very remote villages [39]. This kind of policy aimed to prevent the expression of local (Hutsul) identity by fostering the assimilation of Soviet culture in the Ukrainian territory [40]. Among others, the Soviet regime targeted the expression of Hutsul identity and many traditions and rituals were banned. For instance, wearing Hutsul clothing and singing traditional songs were not allowed [41]. The traditional (religious) calendar was altered and only events devoid of any identitarian features were maintained [41].

The social landscape of Ukrainian Hutsuls abruptly changed in the 1940s when, concomitantly with border creation, drastic depopulation and the collectivization of farms and arable land occurred [42]. Indeed, despite the meagre amount of arable land in the Carpathian valleys, many collective farms were established there, and in the area of Putyla as well (‘There were collective farms and it was hard to live. I have been working since I was 14’, mentioned an elderly individual). Several interviewees reported that there were important wool factories, which benefitted from the large number of sheep present in this area of the Carpathians, in addition to the centralized management of the forest and the promotion of rural clubs (‘Can you believe there was a cinema here?’, asserted a middle-aged male informant).

#### Socio-political factors affecting LEK in Southern Bukovina (Romania)

In Southern Bukovina, beginning in the 1960s, the Romanian government promoted rural systematization (‘sistematizarea’) in order to foster the reconciliation of differences between urban and rural settlements [43]. However, in the following decade, the government recognised the difficulty of rural systematization in the Carpathians, its limited economic potential and the existence of various difficulties, which were sociological, geographical and ethnographical in nature. Therefore, in the 1980s when the main priority turned to agriculture, the project of rural systematization in the Carpathian Mountains was definitively abandoned [43]. In support of this thesis, some local interviewees reported not having experienced the collective farms (otherwise widespread in Romania), due to the limited agricultural productivity of the area. Moreover, local interviewees claimed that livestock and game used to belong to the State, but due to the vastness of the area, the harshness of the steep terrain and communication difficulties, there was not much control in the mountains where Hutsuls live. Therefore, the peripheral location of the area with regard to Romania, as well as its lying along the border and its ethnolinguistic peculiarity prevented this area from being subjected to the centralization policies implemented throughout most of the country (in fact, Romanian Hutsuls reported that only between the 1960s and 1989 were the local forests managed by the central government). As a consequence, ethnobotanical knowledge among Romanian Hutsuls was mainly maintained through vertical transmission (as other sources of knowledge were not widely available).

#### The effects of these different socio-political contexts on medicinal LEK

Therefore, the creation of the border and the consequent socio-political contexts unevenly affected the LEK of Romanian and Ukrainian Hutsuls, despite a common ethnolinguistic background, very similar environmental conditions and the peripherality of these areas in their respective geopolitical contexts. Indeed, in Romania, the area in which Hutsuls live was considered remote and of limited economic interest and as a result left behind in the implementation of the ‘sistematizarea’. In Ukraine, the centripetal power of Moscow was stronger and thus eliminated the concept of peripherality. The reforms were indeed implemented with the same intensity throughout Soviet territory, and the Russian language and collective farms were imposed.

The different success of the policies of the Soviet and Romanian regimes, therefore, differently affected Hutsul LEK. While Romanian Hutsul LEK appears to have been somehow ‘frozen/static’ during the twentieth century, as they were not systematically affected by centralization policies or other factors, Ukrainian Hutsuls were strongly influenced by the new language (Russian) which served as a vector for new (and sometimes technical) knowledge, including the transmission of plant knowledge especially through books and newspapers. Therefore, in addition to vertical knowledge transmission among Ukrainian Hutsuls, we found that other sources of knowledge played an important role. As described in Table 4, these two kinds of LEK sources differ especially with regard to geographical range: while TEK is strictly situational and local and may vary from village to village, other sources may have a wider geographical range, thus encompassing some elements foreign to the community but common to other contexts.

**Table 4:** Characteristics of knowledge sources among Bukovinian Hutsuls

Characteristics	Knowledge mainly orally transmitted	Knowledge in which borders between written and oral forms of knowing nature and practicing this knowledge are more blurred
Language	Mainly local language (Hutsul) but also official languages (Romanian and Ukrainian)	Mainly official languages (Romanian and Ukrainian) but also foreign languages (Russian)
Accessibility	Widely accessible within the village	Not necessarily available within the village.
Geographical range	Strictly situational and local, sometimes varies from village to village	Large geographical ranges (often defined by official language expansion)
Ingredients used	Always local or easily attainable	Not necessarily local

#### Different pathways of medicinal LEK in Northern and Southern Bukovina

Our analysis highlights different trends for food and medicinal LEK among Ukrainian and Romanian Hutsuls. While food uses were quantitatively and qualitatively comparable, about 30% more medicinal uses were reported among Ukrainian Hutsuls. We consider that this might be due to the low availability of physicians and long distances in the sparsely inhabited Hutsul valleys (despite official statistics reporting 3.51 physicians per 1000 inhabitants in Ukraine versus 1.47 in Romania in 1980, [44]), as well as the unavailability of synthetic drugs in health centres.

The higher number of medicinal plants may also be a reaction to Soviet policies which promoted allopathic medicines, discouraging traditional plant-based medicines [35]; for example, a middle-aged Ukrainian women fiercely claimed ‘My mother is 77 years old and has never used a single pill in her life’, and also another women who stated ‘Listen to what is said about medicinal plants so that you do not get sick and do not have to take pills. We drink teas made from Carpathian herbs’. This phenomenon may have been fostered by the severe economic crisis which affected Ukraine after the collapse of the Soviet Union. Indeed, we observed that during this period, medicinal plants were highly promoted by mass media and books; out of the 16 books Ukrainian Hutsuls showed us, 11 were published in this period (1990s).

In the Romania of Ceaușescu, ‘everyone had the right to be hospitalized’, agreed a middle-age couple; however, a younger male interviewee (born in 1974) also reported that ‘at that time (when I was child) there were no doctors, no roads, but there were people who knew plants’, which was confirmed by an older Hutsul woman (born in 1927) who stated ‘when I arrived here (from Ukrainian Bukovina, after border creation), I learnt everything from a local healer and my neighbour. All I knew at the time I came here was the plants we had to harvest for the army during school hours. Among them I remember arnica’. Therefore, it follows that medicinal knowledge in Romania was to some extent ubiquitous, although some local healers held more (maybe also literary) knowledge and were considered reference points within the Hutsul community.

We could not obtain the source of knowledge for each plant, but we can identify some pan-Soviet elements which were not found on the Romanian side of the border. Indeed, we can observe some of the consequences of the reforms implemented in the Soviet era such as the cultivation of *Panax gingseng*, *Ginkgo biloba*, *Aloe vera*, *Aronia melanocarpa* and *Elaeagnus rhamnoides* and their medicinal uses*.* Specifically, *Aronia melanocarpa* gained popularity in the late 1940s when the Soviet Union started large-scale cultivations for the production of juices and jams. However, it was also used as herbal medicine, especially as an antihypertensive and anti-atherosclerotic, in several countries of Eastern Europe including Ukraine [45]. Another example of LEK of pan-Soviet origin is the use of *Elaeagnus rhamnoides*, whose industry, just as with *Aronia melanocarpa*, grew in the 1940s. Its oil was reported in the Russian Pharmacopeia as an anti-inflammatory [46].

As observed by Fedorak [47], despite several changes Bukovina has faced since Austro-Hungarian times, Hutsuls have fiercely strived to maintain their culture, which has been possible, in part, to their scattered dwellings and the remoteness of the mountains. However, the creation of the border resulted in different socio-political circumstances which affected Hutsul LEK in different ways on each side of the border.

Finally, more and more people have resorted to frequenting pharmacies, probably also fostered by globalization and increased economic means (especially among Romanian Hutsuls, who are now European Union citizens). This trend was observed among both Romanian and Ukrainian Hutsuls who often answer to our questions ‘now everyone goes to the pharmacy’.

## Conclusions

We found a total of 118 food and medicinal plants from 107 genera and 53 families. Among Hutsuls of Northern Bukovina we recorded 107 taxa, while there were 72 taxa among Hutsuls of Southern Bukovina. The most used plants were the same in both communities: *Vaccinium myrtillus*, *Rubus idaeus* and *Urtica dioica*.

Despite a common cultural and linguistic background, the ethnobotanical knowledge transmission occurs in different ways on each side of the border. Family is a primary source of ethnobotanical knowledge transmission on both sides of the border; however, in Romania, knowledge from other sources is very limited, whereas in Ukraine interviewees reported several other sources including books, magazines, newspapers, the Internet and television. Indeed, this is especially evident when analysing the wild plants used for medicinal purposes. While recorded food uses are comparable in the two Hutsul communities, our overall data show a disparity regarding the medicinal use of plant taxa. Ukrainian Hutsuls reported around 30% more plant taxa than Romania Hutsuls. The latter group mentioned almost exclusively locally available plants, whereas the former group reported some plants not mentioned by Romanians such as *Aloe vera*, *Maclura pomifera* and *Aronia melanocarpa*. Knowledge regarding these plants was probably not transferred vertically, within the same family, but by other sources of knowledge such as books, newspapers, magazines and possibly radio, as a consequence of the policies implemented during the Soviet era, including the widespread promotion of Russian language and culture, as well as allopathic drugs. Therefore, this may imply hybridization of the local body of knowledge with foreign elements originating in the Soviet context which has enriched the corpus of ethnobotanical knowledge held by Ukrainian Hutsuls.

Further research should specifically address the plant taxa recently introduced in the body of LEK of Ukrainian Hutsuls in order to understand how such knowledge was conveyed and absorbed by Hutsul mountain communities.

## Data Availability

All data are available in this publication.

## Abbreviations

DUR: Detailed use report(s)
JI: Jaccard Index
ICD: International Classification of Diseases
LEK: Local ecological knowledge
RO: Romania
TEK: Traditional ecological knowledge
UA: Ukraine
USSR: Union of Soviet Socialist Republics
WWII: Second World War
